# Mesophilic and Thermophilic Anaerobic Digestion of Wheat Straw in a CSTR System with ‘Synthetic Manure’: Impact of Nickel and Tungsten on Methane Yields, Cell Count, and Microbiome

**DOI:** 10.3390/bioengineering9010013

**Published:** 2022-01-02

**Authors:** Richard Arthur, Sebastian Antonczyk, Sandra Off, Paul A. Scherer

**Affiliations:** 1 Energy Systems Engineering Department, Koforidua Technical University, Koforidua P.O. Box KF 981, Ghana; richard.arthur@ktu.edu.gh; 2 Research Center for Biomass Utilization, Faculty Life Sciences, Hamburg University of Applied Sciences (HAW), 20099 Hamburg, Germany; sebastian_antonczyk@web.de (S.A.); sandra.off@haw-hamburg.de (S.O.)

**Keywords:** straw, trace elements, CSTR, microbiome, cell count, fermentation, biogas, TXRF

## Abstract

Lignocellulosic residues, such as straw, are currently considered as candidates for biogas production. Therefore, straw fermentations were performed to quantitatively estimate methane yields and cell counts, as well as to qualitatively determine the microbiome. Six fully automated, continuously stirred biogas reactors were used: three mesophilic (41 °C) and three thermophilic (58 °C). They were fed every 8 h with milled wheat straw suspension in a defined, buffered salt solution, called ‘synthetic manure’. Total reflection X-ray fluorescence spectrometry analyses showed nickel and tungsten deficiency in the straw suspension. Supplementation of nickel and subsequently tungsten, or with an increasing combined dosage of both elements, resulted in a final concentration of approximately 0.1 mg/L active, dissolved tungsten ions, which caused an increase of the specific methane production, up to 63% under mesophilic and 31% under thermophilic conditions. That is the same optimal range for pure cultures of methanogens or bacteria found in literature. A simultaneous decrease of volatile fatty acids occurred. The Ni/W effect occurred with all three organic loading rates, being 4.5, 7.5, and 9.0 g volatile solids per litre and day, with a concomitant hydraulic retention time of 18, 10, or 8 days, respectively. A maximum specific methane production of 0.254 m^3^ CH_4_, under standard temperature and pressure per kg volatile solids (almost 90% degradation), was obtained. After the final supplementation of tungsten, the cell counts of methanogens increased by 300%, while the total microbial cell counts increased by only 3–62%. The mesophilic methanogenic microflora was shifted from the acetotrophic *Methanosaeta* to the hydrogenotrophic *Methanoculleus* (85%) by tungsten, whereas the H_2_-CO_2_-converter, *Methanothermobacter*, always dominated in the thermophilic fermenters.

## 1. Introduction

Lignocellulosic residues, such as wheat straw or other straw varieties, are currently considered strong candidates to substitute energy crops for low-cost, sustainable biogas and energy production, without competing for food production or land-use change [1]. About 30 million metric tons of cereal straw with 0.8 kg straw/kg grain are produced annually in Germany [2], which represents 14% of the total amount of agricultural residues. Whereas in China, the total output of rice, wheat, and corn straw reached 764 million tons [3]. These agricultural residues are mostly unused or even burnt on the field. When such residues are converted into biogas via anaerobic digestion, the digestate may be returned to the fields as valuable inorganic and organic fertilizer to enhance crop production. The high C/N ratio of wheat straw, of about 80 (Table 1), usually requires a nitrogen-rich co-substrate, such as livestock manure, to prevent process imbalance [1]. In this work, a defined-buffer salt solution was tested over a long-term fermentation to ascertain the exact trace elements requirement of straw during anaerobic degradation. It was adapted to cow manure and was termed ‘synthetic manure’, as shown in Table 2. ‘Synthetic manure’ was used in order to avert unknown inorganic and organic side-effects from cow manure, which is mostly used as co-substrate. Several fermentation experiments with straw were already performed in a continuously stirred tank reactor system (CSTR), but not with a defined medium and automatic intermittent feeding (8 h cycle), similar to a full-scale plant. Furthermore, a high number of publications about trace element supplementation of anaerobic digesters already exists [4,5,6,7], but not with simultaneous monitoring and quantification of the dissolved active, bioavailable trace elements [8,9]. By the presented fermentations, as well as the necessary duration to completely wash the trace elements from the inoculum, should be determined.

Trace elements in environmental samples are usually analysed using inductively coupled plasma optical emission spectrometry (ICP-OES) or inductively coupled plasma mass spectrometry (ICP-MS) after acid digestion of the whole fermenter sample [4,5,10]. However, this turned out to be a disadvantage, because it is not possible to differentiate between precipitated or insoluble elements and dissolved, directly active ions after acid digestion. The situation is further complicated by the fact that carbonates, phosphates, and sulphides precipitate with a very limited solubility in the alkaline pH range of biogas plants, normally being pH 8–8.5 [5,11]. In the presence of hydrogen sulphide, trace elements are actually not bioavailable and exist as insoluble metal sulphides (solubility < 10^−40^ g/L). Hence, there is relatively high uncertainty with the practice of analysing trace elements after acid digestion of whole samples collected from fermenters because the bioavailability is uncertain. It generally requires fastidious empirical assessment and requires several weeks, based on process parameters like volatile fatty acids VFA [12,13]. An alternative method would be to determine the bioavailable trace element levels by using sequential extraction procedure [5,11]. However, this method is time consuming and, therefore, not used frequently. A new method to analyse dissolved, bioavailable trace elements in biogas plants by total reflection X-ray fluorescence spectrometry (TXRF) was developed to overcome this basic problem in a practical manner [8,9].

Cobalt and nickel are the most frequently used trace elements supplements, as they are usually associated with an increase in biogas production; however, distinct single supplementation of tungsten was not reported [5,6,7]. On the contrary, only very small concentrations of tungsten in biogas plants have occasionally been mentioned [4,10]. Two main groups of methanogens can clearly be differentiated using biochemical data, as elaborated in the last 40 years by the Thauer research group [14]. Indeed, trace elements are involved in central catabolic pathways and of paramount importance for anaerobic bacteria and methanogens [14]. Acetotrophic methanogens use only the carbon intermediate acetate, while hydrogenotrophic methanogens consumes H_2_-CO_2_ as a sole energy and carbon source. Firstly, the inert CO_2_ has to be activated by the H_2_-CO_2_-converting methanogens and is incorporated into formic acid (formate) by the unique formylmethanofuran dehydrogenase (FMDH). This catabolic enzyme opens the energy metabolism, and it is very crucial for hydrogenotrophic methanogens but not for acetate converting methanogens [15,16]. Two different FMDHs exist, Types I and II, of which Type I must be induced by molybdenum, while Type II is an enzyme, which contains tungsten [17]. In some cases, tungsten can be used in place of molybdenum [15,18]. However, the essential role of tungsten for H_2_-CO_2_ utilizing methanogens was first observed by [19] using a pure culture of the mesophilic *Methanocorpusculum parvum*, which belongs to the hydrogenotrophic order *Methanomicrobiales* (also including the genus *Methanoculleus*). They used 0.184 mg/L (1 µmolar) radioactive tungsten in the culture medium and observed that tungsten was incorporated into the microbial cells. It led to a strong growth-promoting effect, with an increase in the number of cells by a factor of 5 (measured via the increase of optical cell density) [19]. Astonishingly, this effect occurred even though the culture medium contained very low concentration of molybdenum (0.045 mg/L), which clearly indicated a specific, constitutive tungsten effect [19]. Meanwhile, thermophilic and hyperthermophilic pure cultures of hydrogenotrophic methanogens were found to take up tungsten [17,20], mainly based on enzymological data. Apparently, also some fermentative bacteria need tungsten by their tungsten containing formate dehydrogenase [18,20]. The impact of tungsten and other trace elements on anaerobic digestion and biogas producing, microbial populations have been studied several times [5,6,7], primarily with the most important elements Fe, Ni, Co, Mo, and Se, or a cocktail of them with tungsten, but apparently not tungsten and nickel as distinct, single supplements on a biogas process. Tungsten had generally been supplemented together with several trace elements [5,6,7].

The presented studies were performed with an automated CSTR system using different supplementation-strategies for nickel and tungsten. Wheat straw was used as mono-substrate with different organic loading rates (OLR) and hydraulic retention times (HRT) in a new, defined medium [21,22], both under mesophilic and thermophilic conditions. Quantitative data of microbial cells, together with qualitative microbiome data about major players of microbial classes and genera, provided a complete assessment of anaerobic straw digestion.

## 2. Materials and Methods

### 2.1. Physical and Chemical Properties of Wheat Straw

The wheat straw material (*Triticum aestivum*) used in this study was obtained from a single charge and harvest. It was first shredded to 3 mm with a cutting mill and then gently milled to an average particle size of 0.13 mm using an air vortex mill, which is normally used for milling spices. The air vortex mill was fitted with a vertical turbo reactor and has an air output of around 50,000 m^3^/h at a product temperature of around 40–50 °C (Görgens Mahltechnik, Dormagen, www.mahltechnik-goergens.de, accessed on 15 January 2021). The biochemical and elemental composition of the milled straw (Table 1) was determined, in order to obtain information about the degradable and non-degradable components of the wheat straw, as well as to calculate the theoretical, maximum biomethane potential (BMPth), using the extended chemical Buswell formula of Boyle, as outlined by author [23].

Biochemical constituents, elemental CHNOSP, and Klason lignin analyses (Table 1) were performed at the Institute for Wood Chemistry of the von Thünen Society, Hamburg-Bergedorf, according to [24].

### 2.2. Synthetic Manure as Fermentation Medium

The composition of the ‘synthetic manure’ was based on a long-term chemical analysis of cattle manure from a dairy farm [21], the cattle were fed with maize and grass. Thereafter, ‘synthetic manure’ was prepared and used for the continuous fermentation experiments, under mesophilic (41 °C) and thermophilic (58 °C) conditions [22] (Table 2). The alkalinity of the natural cattle manure was found to be in the range of 8000–12,000 mg CaCO_3_ eq./L, whereas that of the prepared ‘synthetic manure’ was 10,865 ± 330 mg CaCO_3_ eq./L. The resulting pH lay between 7.3–7.4 (mesophilic) and 7.4–7.6 (thermophilic).

### 2.3. Measurement of Offline Parameters

#### 2.3.1. Process Control Parameters

The concentration of VFA (C_1_–C_6_) was estimated weekly using GC, as described under lab 11 in [25] in a 25-interlaboratory study. The plotting time for VFA was 10 min, while the phenyl acetic acid was released from the GC column after 25–30 min. However, the phenyl acetic acid values were not recorded, as performed by [26], because unlike the VFA, they seemed not to be relevant process imbalance indicator in this study. Buffer capacity (alkalinity TAC or TIC) was estimated according to [27], as outlined by Scherer et al. [12]. Salt load (conductivity), total solids = dry weight (TS), was determined by drying the sample 8 h at 105 °C to a constant weight, as well as volatile solids = organic dry weight (VS) or ash-free organic dry matter by heating 5 h at 540 °C to a constant weight. Phosphate (PO_4_^3−^) was estimated using a photometric cuvette test from Macherey and Nagel (No 91878, Düren, Germany), whereas ammonium (NH_4_^+^) was determined with a photometric cuvette test from Merck (No 1.00683, Darmstadt, Germany). The ammonia concentration was calculated using the equation according to [28].

#### 2.3.2. Trace Elements Analysis

Potassium, calcium, manganese, iron, cobalt, nickel copper, zinc, selenium, and tungsten were determined every fortnight (in some periods weekly) using total reflection X-ray fluorescence spectrometry (TXRF), which is mainly suitable for heavy elements analysis (mol weight > 50), according to recent studies [8,9]. The methods [8,9] were developed to enable easy quantification of trace elements in their dissolved, active forms, as only ions can be taken up through the microbial cell membrane. TXRF is applicable for liquid and dry samples. In this work, the bench-top instrument, S2 PICOFOX^®^ from Bruker (Bruker Nano GmbH, Berlin, Germany), was used as it operates with a molybdenum (Mo) tube as the detector, instead of a silver (Ag) tube detector. This setup resulted in a 10-fold higher sensitivity (detection limit about 0.002 mg/L), but it was not possible to quantify Mo [29]. However, the restriction made it possible, for the first time, to study the anaerobic degradation of straw on the basis of free available ions.

#### 2.3.3. Microbial Analysis

The methanogens were quantitatively determined using their characteristic auto-fluorescence, whereas the total microbial cell counts were estimated based on SYBR GreenI [30], to avoid counting of undigested straw particles in the process. Furthermore, the total cell counts were enumerated with the help of a photo-microscope. The pictures taken were quantified by image analysis, as developed in [30,31]. The critical aspect of the analysis was the preparation of a single cell layer on a glass slide and estimation of the total area of irregular-shaped samples. The sensitive digital microscope camera Leica DFC 365 FX, in combination with the Leica DM600B fluorescence microscope and software Image Pro 7, was used. Leica CFP with excitation 426–446 nm and emission 460–500 nm was the filter applied for the analysis of methanogens. On the other hand, SYBR green-stained total cells were counted with the Leica L5 filter set, with an excitation of 460–500 nm and emission of 512–542 nm.

### 2.4. Next Generation Sequencing Data (NGS)

The DNA of the microbial community was extracted using the MP Biomedicals™ FastDNA™ SPIN kit, mostly used for soil, while the mechanical cell disruption was performed twice at 6500 rpm for 30 s using the Precellys^®^ 24 homogenizer (Peqlab, Germany). Intensive mechanical disruption was found to be crucial, in order to obtain the total number of methanogens. Finally, the samples were purified using the Genomic DNA Clean & Concentrator kit (Zymo Research, Irvine, CA, USA) to recover pure DNA.

The 16S rRNA gene amplicon sequencing was performed by LGC Genomics (LGC Genomics GmbH, Berlin, Germany) using 300 bp paired-end reads, as well as by sequencing the V3 and V4 region on an Illumina MiSeq sequencer (Illumina, Inc., San Diego, CA, USA). The primers Pro341F (5′-CCTACGGGNBGCASCAG-3′) and Pro805R (5′-GACTACNVGGGTATCTAATCC-3′), covering the domains bacteria and archaea, were used for the first PCR round [32].

Based on the protocol from LGC (LGC Genomics GmbH, Berlin, Germany), for amplicon processing, libraries for each sequencing lane were demultiplexed by Illumina bcl2fastq 2.17.1.14 software. At least 250,000 reads per sample were obtained. Raw reads were sorted by amplicon inline barcodes and adapters were clipped. Reads with a final length <100 bases were discarded. In addition, primer sequences were detected and clipped, allowing three mismatches per primer. Subsequently, high-quality sequences were merged using BBMerge 34.48 (http://bbmap.sourceforge.net/, accessed on 1 November 2021).

The 16S pre-processing and operational taxonomic unit (OTU) clustering from amplicons were performed with the program Mothur 1.35.1, as follows: (a) removal of sequences containing ambiguous bases, with homopolymer stretches of more than 8 bases or with an average Phred quality score below 33, (b) alignment against the 16S Mothur-Silva SEED r119 and, additionally, against the Silva SSU r132 database version from 2019 and reference alignment, (c) filtering of short alignments (truncated or unspecific PCR products), (d) sequencing error reduction by pre-clustering (up to 1 differing base per 100 bases allowed in a cluster), (e) elimination of chimera with the uchime algorithm, (f) taxonomical classification of the sequences (against the Silva reference classification) [33] and removal of sequences from other domains of life, (g) OTU picking by clustering at the 97% identity level (using the cluster.split method), and (h) OTU consensus taxonomical calling, integrating the taxonomical classification of the cluster member sequences. Finally, these processing steps resulted in the creation of an OTU count table showing taxonomic distributions.

The representative sequences of each OTU (operational taxonomic unit) were queried against the ribosomal database project release 11.4 reference. Annotations of the putative species levels of the OTUs was performed with the NCBI BLAST + 2.2.29 (basic local alignment search tool). The raw amplicon sequencing data of this study were deposited in the short read archive (SRA), under the bioproject ID PRJEB48733.

### 2.5. Continuously Operated Laboratory Fermenters

**Figure 1:**
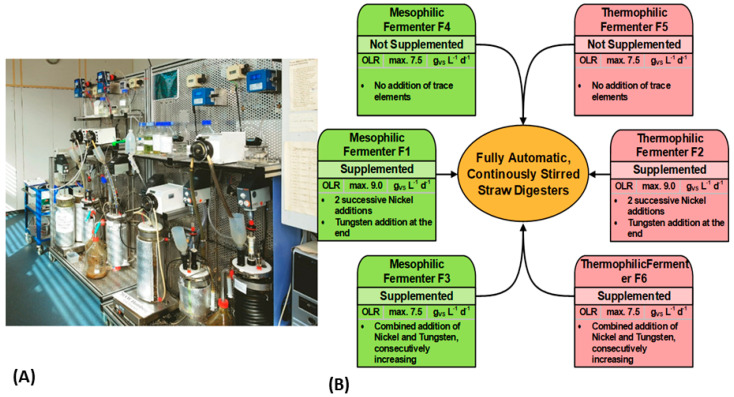
(**A**) A view of the continuously operated and fully automated 6-L laboratory anaerobic fermenters, developed and built at the university campus in Hamburg-Bergedorf. (**B**) Graphical summary of the experimental design for wheat straw digestion with ‘synthetic manure’, over a period of nearly 3 years. Investigation of temperature influence, OLR, and trace element requirements, during mesophilic (41 °C, green) and thermophilic (58 °C, red) fermentation. The following values of OLR and HRT were adjusted: low OLR 4.5 (HRT 18 days), medium OLR 7.5 (HRT 10 days), and high OLR 9.0 gVS/L/d (HRT 8 days). Two trace element supplementation strategies were implemented: 1. Successive increment of nickel (Ni) in 4 periods with different OLRs, then final addition of tungsten (W) in period 5 in fermenter F1 and F2. 2. Combined addition of Ni and W in fermenter F3 and F6 in three periods with the same OLR: from the beginning with a low sum ∑(Ni + W), followed by an increased sum ∑(Ni + W). F4 and F5 were reference fermenters, without trace element supplementation.

Six automated 6-L (liquid volume), continuously-operated laboratory glass fermenters were used for this work (Figure 1). The fermenters had simple outlets through overflow and were originally inoculated with 1/3 liquid swine manure, 1/3 sewage sludge, and 1/3 hot rot compost suspension by volume, to achieve a rich variety of microorganisms in the beginning [34,35]. Three fermenters of the same temperature were connected to a single cooled substrate storage tank (4 °C). Coiled tubes filled with water were wrapped around the fermenters for heating or cooling, depending on the required temperature. The fermentation temperatures, 41 °C and 58 °C, as well as cooling of the substrate tank (4 °C), were established using Lauda instruments, i.e., E100 and RE107 (Lauda-Königshofen, Germany). Each fermenter was equipped with the stirrer Heidolph RZR 2020 (Heidolph Instruments, Schwabach, Germany) and a peristaltic pump GUV-150 (Meredos GmbH, Bovenden, Germany). They were placed on an electronic balance, Sartorius E_B35EDEI (Sartorius AG, Göttingen, Germany).

An amount of 1 kg of the milled straw was added to 10 L of ‘synthetic manure’ to obtain a 10.15 L straw suspension and stored at 4 °C. Apparently, the straw or the bacteria did not swell in the aqueous liquid. However, it was observed that suspending the milled straw in liquid made the substrate easier to handle for this laboratory scale experiment. The fermenters were fed every 8 h with fresh substrate through an intermittent mode using a peristaltic pump, similar to the feeding routine by [34] and full-scale biogas plants. However, in the present work, the fermenters were continuously stirred at 150 rpm. The paddle stirrer had an additional side wing directly above the bottom of the fermenter and one directly below the liquid level. That prevented the formation of sediments and scum.

**Figure 2:**
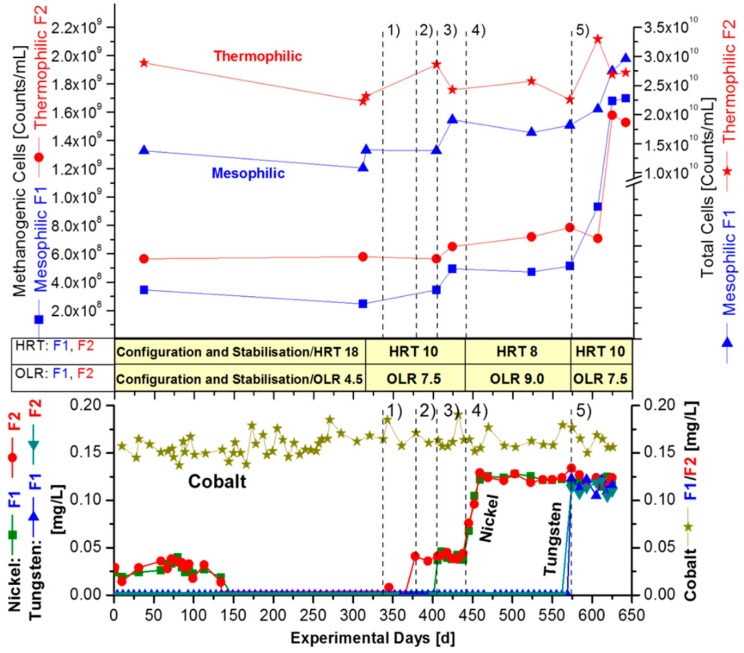
Successive addition of nickel in mesophilic and thermophilic CSTR fermenters, with wheat straw as the sole substrate in ‘synthetic manure’. Dissolved ions in fermenter F1 (mesophilic) and F2 (thermophilic). Targeted concentrations of supplemented and monitored ions of nickel and tungsten were: Period 1 (340–370 d) + 0.05 mg/L Ni in F2. Period 2 (370–405 d) + 0.05 mg/L Ni in F2. Period 3 (405–440 d) + 0.05 mg/L Ni in F1, F2. Period 4 (440–570 d) + 0.15 mg/L Ni + EDTA in F1/F2. Period 5 (570–640 d) + 0.15 mg/L Tungsten in F1/F2 (without EDTA). Dissolved ion concentrations of cobalt, nickel, and tungsten, as analysed with TXRF. A pre-fermentation period of about 140 days, with an HRT of 18 days and OLR of 4.5 gVS/L/d was performed ahead (not shown here) to enable technical modifications.

**Figure 3:**
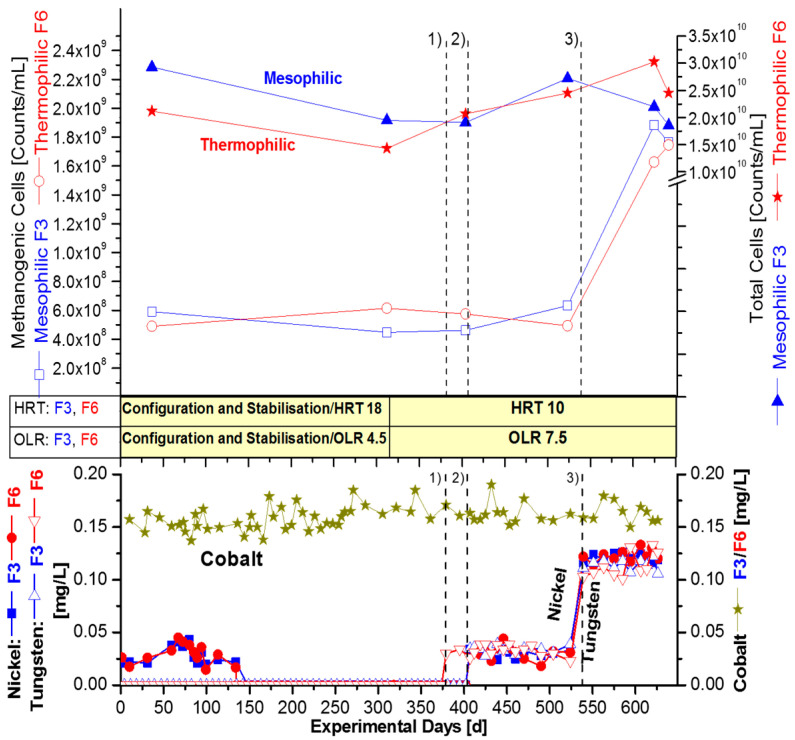
Combined addition of [nickel + tungsten] in fermenter F3 (mesophilic) and F6 (thermophilic) over 640 days in 3 fermentation periods, with wheat straw as the sole substrate in ‘synthetic manure’. The OLR was constant 7.5 gVS/L/d with the HRT of 10 days. Targeted concentrations of supplemented and monitored ions of nickel and tungsten were: Period 1 (370–405 d) + 0.05 mg W/L, only in F6; Period 2 (405–540 d) + 0.05 mg/L [Ni + W] + EDTA, both in F3 and F6; Period 3 (540–640 d) + 0.15 mg/L [Ni + W] + EDTA, both in F3 and F6. Concentrations of dissolved ions of cobalt, nickel, and tungsten as analysed with TXRF. A pre-fermentation period of about 140 days, with an HRT of 18 days and an OLR of 4.5 gVS/L/d was performed ahead (not shown here) to enable technical modifications.

**Figure 4:**
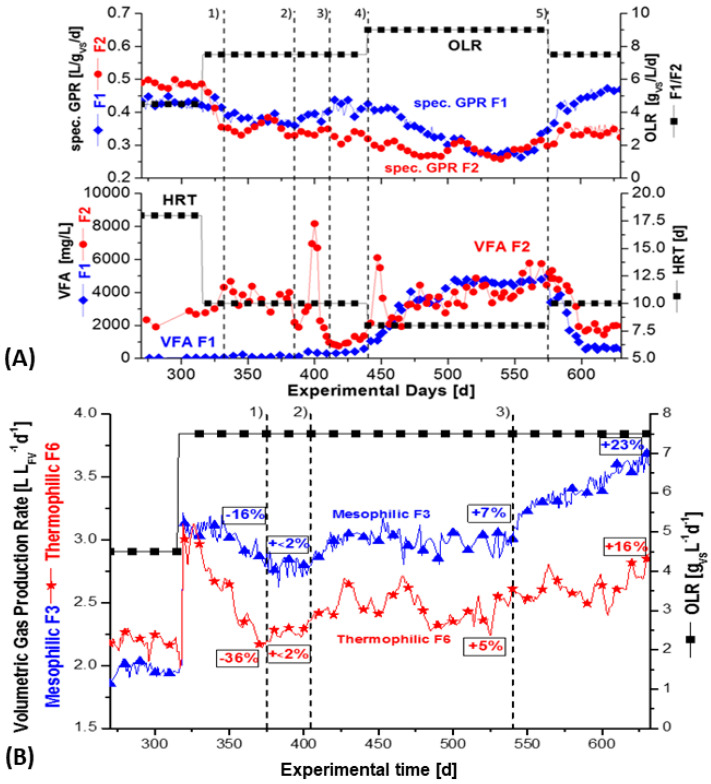
(**A**) Specific biogas production (L_STP_) per gram VS wheat straw of fermenter F1 and F2 during the successive addition of nickel, further addition of EDTA, and, finally, tungsten in fermentation period 5. Supplementation of tungsten in period 5 resulted in tripling of the population of methanogens. The OLR was changed during the fermentation periods, as indicated and according to Figure 2. (**B**) Volumetric biogas production in fermenter F3 and F6, with constant OLR, as indicated in Figure 3. Gradual increase of nickel and tungsten during combined supplementation, with low ∑(Ni + W) at the beginning, followed by high ∑(Ni + W) at the end. Blue: mesophilic; red: thermophilic; 51.5% CH_4_ in biogas.

The bivalent NiCl_2_·6 H_2_O and the trivalent Na_2_WO_4_·2 H_2_O were used as trace element supplements. The dosage was adapted to the trace elements washout caused by the different HRTs. Therefore, a short HRT required a heightened Ni- or W-ion supplementation to get the same concentration in the fermentation liquid. Before feeding, the tubes were purged by reverse pumping, in order to avoid uncontrolled fermentation in the tube during the 8 h intervals. During feeding, an electronic valve was opened to let off the overpressure and disable erroneous gas measurements. Additionally, the amount of fresh substrate, based on a given OLR, was predetermined and programmed into the control software developed in-house. A pre-fermentation period of about 140 days with an HRT of 18 days and OLR of 4.5 gVS/L/d was performed ahead to enable technical modifications (not shown in Figure 2, Figure 3 and Figure 4). The subsequent experimental period was graded by experimental days.

### 2.6. Measurement of Online Parameters

Online measurements were performed every 10 min, then an average value was calculated every 60 min, which was summarized at the end of every 24 h. The pH value in all six fermenters was measured with a Xerolyte electrode HA405-DXK-S8/225 (Mettler-Toledo in Giessen, Germany). Additionally, Xerolyte electrode Pt-DXK 4805-S8/225 (Mettler-Toledo in Giessen, Germany) was used to measure the redox values (reduction-oxidation potentials). Originally, the intention was to use the redox value for regulating the feeding rate, to prevent an increase of the VFA level. The recorded redox value was more positive, when the VFA increased. However, this behaviour occurred only in weakly buffered media and not in the ‘synthetic manure’. Furthermore, the CH_4_ and CO_2_ concentrations were measured using BCP-CH_4_ and BCP-CO_2_ sensors (BlueSens GmbH, Herten, Germany), respectively. The volumetric biogas production was measured using a bubble counter and calibrated tilting body with two 10 mL, instead of 1 mL chambers, MilligasCounter^®^, type MGC 10, equipped with a condensed water trap (from Dr. Ritter GmbH, Bochum, Germany). The barrier liquid in the MGC was 0.1 molar HCl, as proposed by [36]. Gas volumes were “standardised”, according to IUPAC, with the standard temperature 0 °C and standard pressure 1 bar (STP). For further details, see [22].

### 2.7. Statistical Analysis

The data obtained from the calculated average of the specific biogas yields and methane production yields for all the experiments were subjected to analysis of variance (ANOVA), in order to statistically determine the significant differences in the results [37]. The term significant was used only when a statistical test was performed using a *p*-value < 0.05.

## 3. Results and Discussion

### 3.1. Trace Element Analyses of Straw Fermentation Medium with ‘Synthetic Manure’

Gradual purging of the trace elements in the inoculum sludge, technical modifications, and process stabilization occurred in the start-up phase of about 140 days and an HRT of 18 d (see Section 2.5), which was continued after the commencement of the study to 340 days in Figure 2 and 370 days in Figure 3. Simultaneously, the monitoring for the fermentation campaign of the three mesophilic (41 °C) and three thermophilic (58 °C) fermenters was executed, which lasted for 640 days. The objective was to determine the trace element requirement for straw digestion, as well as maximum methane yields under defined conditions. The analysis of the straw, as shown in Table 1, indicated that there was sufficient cobalt concentration in the wheat straw substrate. This is also demonstrated by the continuously monitored dissolved cobalt concentration in the fermenters (Figure 2 and Figure 3 and Table 3). However, tungsten supplementation was required, as it was estimated to be below detection limit in the dry mass, Table 1. The appreciable nickel concentration of 0.58 mg/kg in dry matter (Table 1) is a good example to show that the elemental content of dried substrate (with or without fermentation medium) could be deceptive. It was later found that the amount of dissolved nickel was not sufficient for the entire fermentation period, see Figure 2 and Figure 3. Therefore, supplementation with these elements was considered in the fermentation experiment. Furthermore, the safe limit of OLR or HRT for straw digestion under mesophilic and thermophilic conditions was evaluated, according to the fermentation program in Figure 1B. The following terms for OLRs were used: 4.5 as ‘low’, 7.5 as ‘medium’, and 9.0 gVS/L/d as ‘high’. The resulting HRT was diminished congruently by the OLR to 18, 10, or 8 days, respectively, because it was coupled with the liquid 10% straw suspension as substrate.

The molecular formula of the wheat straw used was found to be C_3.71_H_6.04_O_2.79_N_0.044_S_0.005_ (Table 1). Based on this formula, the theoretical maximum BMPth was calculated to be 293.4 mL_STP_/gVS straw, with a theoretical methane content of 51.8% in the biogas (measured 51.5%). To obtain the index values of the molecular formula, the percentage-content of the elements (Table 1) were divided by the corresponding atomic number of C, H, O, N, S, and P.

The alkalinity of the six parallel fermenters were in the range of 7000 and 9600 mg CaCO_3_-eq./L during the fermentation, as it was influenced by the OLR. Therefore, the buffer capacity of the fermentation medium was marginally lower than that of the ‘synthetic manure’ (Section 2.2 and Table 2), but the buffer capacity was almost always twice the value (4000 mg CaCO_3_-eq./L) required to guarantee a pH of around 7.0 [38]. The resulting pH value was between 7.3–7.4 (mesophilic) and 7.4–7.6 (thermophilic). There was an excess phosphate from the straw substrate, because the phosphate concentration increased from about 140 mg/L up to 260 mg/L during the entire fermentation period.

The ammonium concentration of the fermentation media was controlled mainly by the defined ‘synthetic manure’ (Table 2), it was about 1940 mg/L or 108 mmol L^−1^ [22]. The highest ammonia value obtained was 800 mg/L for the thermophilic fermenter F5. Its concentration was mainly influenced by the different temperatures of the fermenters, according to [28], as the pH was stable (Figure 2, Figure 3 and Figure 4). However, even at this level of 800 mg/L, there should be no inhibition, as the cultures adapted and acclimatized well. This assumption was based on the recent review by [39] because thermophilic systems with temperatures around 55 °C can carry out efficient anaerobic digestion, without any sign of inhibition, at ammonia concentrations up to 1000–1500 mg/L.

Table 3 shows the concentration of the dissolved ions of Mn, Fe, Co, Cu, Zn, Ni, and W during the 640 days of fermentation. The potassium cation was chosen as a reference ion, as it does not form precipitates with the common anions. Calcium was taken as the second reference ion because it can precipitate as carbonate. Both reference ions are light elements with atomic number of 19 and 20, respectively, and can determined with TXRF with uncertainty. Interestingly, in the case of calcium, less than half of the concentration, being present in the mesophilic fermenters, was estimated in the thermophilic fermenters. No significant difference was seen for cobalt, copper, and zinc ions, with regard to the different fermentation temperatures. Low dissolved ions concentration of around 0.05 mg/L was found during the fermentation process. However, dissolved nickel and tungsten concentrations were below the detection limit (less than 0.002 mg/L [9]) under both temperature conditions. There was only a small difference between the iron concentration of all the six fermenters, as the average concentration was about 9.1 for mesophilic and 7.8 mg/L for thermophilic fermenters, respectively (Table 3).

Nonetheless, it was obvious that the concentration of manganese ions in the thermophilic fermenters was significantly higher than in the mesophilic fermenters. The selective character made sedimentation an unlikely cause. Therefore, the observed six times less manganese concentration in the thermophilic fermenters could mean that there was a higher consumption of manganese by the thermophilic microbial population or even thermophilic methanogens (Table 3). So far, data (with respect to this phenomenon) have not been found in literature. Furthermore, data from literature generally refer to the whole fermenter content, including substrate and digestate, as well, and do not differentiate between dissolved or undissolved precipitations. The six-fold, relatively low concentration of manganese in the thermophilic fermenters for the methanogenic population remains a phenomenon, which could possibly be investigated in a separate study.

### 3.2. Requirement of Nickel and Tungsten for Ultimate Digestion of Wheat Straw

After a fermentation period of 150 days (with an additional pre-phase of 140 days, see Section 2.5), a complete ‘washout’ of all relevant trace elements from the seed sludge could be observed, as the nickel concentration dropped down below the detection limit BDL. The concentration of dissolved nickel ions in mesophilic fermenter F1 and thermophilic fermenter F2 decreased simultaneously from an initial 0.040 mg/L to less than 0.002 mg/L (BDL) after 150 days of elution (Figure 2). Exactly the same effect was observed in F3 and F6. Tungsten was not detected from the beginning of the fermentation campaign in the straw substrate (Table 1) or in the fermentation medium (Table 3, Figure 3). The dissolved cobalt concentration always lies around 0.150 mg/L in each fermenter and was, therefore, sufficient. Thus, it was not necessary to supplement the fermenters with cobalt over the whole fermentation campaign (Figure 2 and Figure 3). The additional pre-phase of 140 days was performed with an HRT of 18 days for technical modifications and stabilization. The entire monitored fermentation period lasted 640 days (Figure 2 and Figure 3). After complete washout of trace element from the inoculum, the actual trace element supplementations were initiated in the mesophilic (F1/F3) and thermophilic (F2/F6) fermenters. Two strategies were applied:

i. Successive increment of nickel (Ni), in four periods and with different OLRs; then, a final dosage of tungsten (W) in period 5 in fermenter F1 and F2 (Figure 2).
ii. Combined addition of nickel and tungsten, in fermenter F3 and F6, in three periods with equal OLR. It was started the combined supplementation by a low sum ∑(Ni + W), followed by an increased sum ∑(Ni + W) (Figure 3).

#### 3.2.1. Successive Supplementation of Nickel Plus Tungsten

F4 and F5 were used as references fermenters without trace element supplementation. The first OLR was set to 4.5 gVS/L/d, coupled with an HRT of 18 days, which was then increased to OLR 7.5 gVS/L/d (HRT of 10 days) from fermentation day 340 to 405.

After the washout phase of residual trace elements, the thermophilic fermenter F2 was supplemented in period 1 on day 340, to obtain the targeted concentration of 0.05 mg/L of dissolved nickel ions. Afterwards, also the mesophilic fermenter F1 was supplemented with nickel in the same manner, but that started in period 3 (405–440 d). However, with the target of 0.05 mg/L, the measured dissolved nickel concentration provided only 0.040 mg/L (Figure 2).

There was no indication of VFA stress in the fermentation periods 1–3 of F1 and F2, as shown in Figure 4A, Table 4 and Table 5. Thus, the concentration of nickel supplementation was increased to 0.15 mg/L in periods 4 (440–570 d) and 5 (570–640 d). Meanwhile, on fermentation day 440, EDTA, as complexing agent for bivalent ions, was added to the same concentration of nickel in the 4th fermentation period. A similar kind of control-experiment was previously reported by [40]. They found a stimulating effect of EDTA in the presence of the bivalent nickel on the specific methane yield of a synthetic substrate consisting of a mixture of VFA. In their work, the EDTA almost tripled the solubility of the nickel from 0.040 mg/L to 0.125 mg/L, which presumably was the reason for the positive effect observed by [40]. EDTA is a general complexing agent for bivalent ions, but the supplemented Na_2_WO_4_·2 H_2_O is trivalent. Accordingly, increasing effects of EDTA on tungstate have not been reported.

Furthermore, the strategy was to increase the OLR, in order to push the requirement of trace elements. Therefore, the OLR was increased in period 4 from 7.5 to 9.0 gVS/L/d. However, apparently, this increase of OLR, with decrease of HRT, could mean a process imbalance, as the concomitant reduction of HRT from 18 to 10–8 days resulted in a simultaneous increase of VFA in F1 and F2 between fermentation day 440–540 d. The VFA level increased to about 4000 mg/L under mesophilic and 6000 mg/L under thermophilic fermenter conditions, F2 (Figure 4A, Table 4 and Table 5). The estimated VFAs were found to be mainly acetic acid >80%, <15% propionic acid, and about 2.5% butyric acid. VFAs are regarded as a very sensitive indicator, and the occurrence of VFAs >1000 mg/L is generally taken as a sign of process imbalance [12,13]. Especially at low pH, the uncharged but membrane permeable VFA molecules are formed. Therefore, a constant pH level should generally be considered a fundamental parameter for microbes. As outlined, the buffer capacity of the composed ‘synthetic manure’ (Table 2) prevented a pH imbalance during fermentation. The pH remained stable between 7.3 and 7.4 in the mesophilic fermenters, whereas it stayed between 7.4 and 7.6 in the thermophilic ones. This was monitored for the whole fermentation campaign. Additionally, the redox potential was found to be relatively constant during the fermentation phase of 290 days, as well as the other periods, with around −330 ± 10 mV at 41 °C and at −350 ± 10 mV at 58 °C.

The stable pH conditions in this work are similar to the situation in large-scale biogas plants, characteristic of having high amounts (>60%) of natural manure and other types of substrates. However, to prevent and overcome any risk of an imbalance, the OLR in period 5 (570–640 day) was reduced from 9.0 to 7.5 gVS/L/day (Figure 2). In contrast, the observed increased cell counts revealed that the simultaneous rise in VFA caused no real instability (Table 5). This new insight, with respect to the number of microbial cells, contradicts the general operating experience [12,13] and illustrates that the sensitive VFA level should be not over-interpreted for process imbalance. The successive nickel addition in periods 1–3 (fermentation 370–540 day), with an HRT of 10 days, resulted in the rise of methanogenic cells from 2.5 × 10^8^ to 4.9 × 10^8^ cells per mL in the mesophilic fermenter F1, as well as from 5.8 × 10^8^ to 6.5 × 10^8^ in the thermophilic fermenter F2 (Table 5, 311 and 423 day).

Thus, corresponding to the addition of only traces of nickel, an increase of the methanogenic population, by a factor of almost 2 in F1 (mesophilic) and by a factor 1.1 in F2 (thermophilic), resulted. Even with the strongly reduced HRT of 8 days in the fermenters F1 and F2 in period 4, the number of methanogenic cells continued to increase by 9% between fermentation days 521–570 day in both fermenters, with quite different temperatures. However, the total cell counts of attendant thermophilic bacteria decreased somewhat by 12.5% in this period 4 (Table 5). Seemingly, the thermophilic population was more sensitive to changes in process parameters. Tungsten was still below the limit of detection (<0.002 mg/L) in fermentation period 4. Therefore, an amount of 0.15 mg/L of tungsten was added in period 5 (fermentation day 570–640, but without addition of EDTA) and triggered a dissolved concentration of 0.125 mg/L. Indeed, as assumed, the methane production went up and, at the same time, an obvious decrease of the VFA-level occurred, with a change from 4000 to about 1000 mg/L in the mesophilic fermenter F1. In case of the thermophilic fermenter F2, the VFA declined from 6000 to 2000 mg/L after tungsten was added in period 5 (Figure 4, Table 5). This phenomenon is a further indication that the hydrolytic and acid-producing bacteria functioned effectively and was well-balanced under mesophilic, rather than under thermophilic conditions, with the methanogenic part of the anaerobic food chain. For further information about the food chain see the review [41]. However, this statement is related to straw fermentation with a high or very high OLR, 7.5 or 9.0 gVS/L/day, respectively, and with a corresponding short HRT of 10 or 8 days (Figure 4).

However, adding tungsten after nickel was supplemented, again, caused a tremendous enhancement of the number of methanogens in the mesophilic fermenter, F1 by a factor of 3.3 or 330% (from 5.1 × 10^8^ up to 1.7 × 10^9^ cells per mL), and in the thermophilic fermenter, F2 by a factor of 1.9 (from 7.9 × 10^8^ to 1.5 × 10^9^ methanogens/mL), which occurred in fermentation period 5 (570–640 day) after tungsten addition (Figure 2, Table 4). However, the total cell counts after tungsten addition increased by only 62%, from 1.8 × 10^10^ to 3.0 × 10^10^ (mesophilic fermenter, F1) and by 20% from 2.3 × 10^10^ to 2.7 × 10^10^ (thermophilic fermenter, F2), within period 5 from fermentation days 570–640 (Figure 2, Table 5). This striking increase in the number of methanogens, but not of total cells, is a strong indication that tungsten affected the methanogens, specifically. Therefore, it can be concluded that, apart from the other trace element levels presented in Table 3, dissolved ions of nickel and tungsten, with concentrations of around 0.1 mg/L, were required for an efficient and stable anaerobic degradation of straw, to ensure low VFA-levels (Figure 2, Figure 3 and Figure 4).

#### 3.2.2. Combined Supplementation of Nickel Plus Tungsten

The supplementation strategy in Figure 3 was different from Figure 2, because in Figure 3 simultaneous supplementation of nickel and tungsten was conducted, instead of a successive increase of nickel dosage, with the final adding of tungsten. The modified supplementation strategy for the mesophilic fermenter F3 and thermophilic fermenter F6 was bundled in three longer fermentation periods, from 380–640 day, with a uniformly constant high OLR of 7.5 gVS/L/d and concomitant HRT of 10 days. However, the same final concentrations of nickel and tungsten were adjusted, as presented in Figure 2, with successive supplementation.

The series of combined supplementations were started with the dissolved nickel concentration of 0.040 mg/L in the thermophilic fermenter F6, although the supplemented concentration was 0.050 mg/L (see 370 day, period 1 in Figure 3). The mesophilic fermenters F3 was supplemented in the subsequent fermentation period 2, on 405 d, as well as the thermophilic fermenter F6, with the same concentration of nickel, such as the one with EDTA.

Finally, in fermentation period 3 (starting on 540 day), the combined nickel and tungsten supplementation concentration was tripled to 0.150 mg/L, but the concentration of dissolved ions was 0.125 mg/L for both trace elements, Figure 3. This revealed exactly the same final concentration of 0.125 mg/L, as already measured during fermentation in Figure 2, but with a different OLR regime of varying OLRs and by successive addition of Ni and W, instead of the combination. Apparently, different OLR did not change the demand of nickel or tungsten. The explanation for this phenomenon seems to be due to the absence of nickel and tungsten in the straw substrate and possibly a necessary threshold concentration (Table 1).

It is noteworthy, that a similar increase in the number of methanogens was observed after the combined addition of nickel and tungsten (Figure 3), if compared with the successive supplementation of nickel, as well as after it by addition of tungsten in a final step (Figure 2). Methanogens in the mesophilic fermenter F3 rose by a factor of 3.6 (360%) from 4.9 × 10^8^ to 1.8 × 10^9^ methanogens/mL. Remarkably, they rose equally, by the same factor of 3.6 from 4.9 × 10^8^ to 1.8 × 10^9^ methanogens/mL in the parallel thermophilic fermenter F6 (between fermentation day 37 and 640 day, Table 4).

The impact of nickel and tungsten on the total number of cells turned out to, again, be much lower with the combined dosage of nickel and tungsten. The total cell counts increased by only 3% from 2.1 × 10^10^ to 2.2 × 10^10^ cells/mL in fermenter F3 and by 42% from 2.1 × 10^10^ to 3.0 × 10^10^ total cells/mL in fermenter F6 (Table 5). The cell numbers of Table 5 clearly indicate that the nickel and tungsten effect was predominantly related to the population of methanogens, but not at the same level of the accompanying bacteria being hydrolytic or syntrophic.

The low proportion of 4–9% methanogens (maximum 1.76 × 10^9^ per mL), relative to the total cell counts (maximum 3.29 × 10^10^ per mL, Table 5), is not unusual, but with similar relations typical of complex substrates in agricultural biogas plants [30,31,32].

#### 3.2.3. TS/VS Content of the Anaerobic Straw Digesters

All the straw digesters, mesophilic or thermophilic, were supplied with the same inoculum (see Section 2.5) but were still in continuous operation for more than 10 years before the new start [34,35]. Microscopic observations indicated that the TS contents of the six fermenters consisted mainly of sedimented, undigested straw particles (digestate). Therefore, in addition, besides single cell counts, the VS content was also monitored continuously in all straw digesters. It represented a rough sum parameter for growth and undigested straw particles. The initial TS of the fermentation campaign was estimated to be an average of 4.4% (70% VS). At the end, almost the same final TS content of 7.3% (82% VS) was found on average for all fermenters, including the unsupplemented reference fermenters F4 and F5. Therefore, the maximum recorded total cell counts of 3.0 × 10^10^ cells/mL (F1, fermentation day 640 day, Table 6) did not directly reflect the TS or VS content of the fermenters, due to the low content of microbial cell mass, which is about 10^−12^ g per single cell (assumed cell size with a cube 1 µm × 1 µm, density 1). They would contribute a maximum of about 0.3% TS during straw fermentation and only 4% of the final TS-fermenter content. Based on these physical properties, a pure aqueous bacterial cell culture cannot contain a higher cell density than general 10^10^–10^11^ cells/mL. In reference to that, the estimated number of microbial cells in the used laboratory fermenters were in the same range (Table 5). However, surprisingly, cell numbers and VS/TS-values in large-scale agricultural biogas plants were of the same order, as found here [30,32]. Therefore, it follows that cell growth in the laboratory CSTR system behaved like in a full-scale, solid-state fermentation. Consequently, the obtained results from the laboratory CSTR system could serve as a basis for the layout of full-scale plants.

### 3.3. Methane Yields of Anaerobic Straw Digestion

A trend of the specific methane gas production (mL_STP_ CH_4_/gVS) in fermenter F1 (mesophilic) and F2 (thermophilic) is presented in Figure 4A, to show the efficiency of substrate conversion into methane. Alternatively, in Figure 4B, the volumetric biogas production (sum of CH_4_ + CO_2_, with CH_4_ being 51.5%) of fermenter F3 (mesophilic) and F6 (thermophilic) is pictured to demonstrate more the dynamics of gas production during mesophilic and thermophilic straw fermentation (L biogas/L fermenter volume FV).

Fermenters F1 and F2 were successively supplemented with nickel in four periods and then, finally, supplemented with tungsten, as shown in Figure 2. In the first fermentation period until 330 d (Figure 2), a mixed situation with an incomplete washout of trace elements occurred, the highest specific GPR was observed for the thermophilic fermenter F2 and F6 at around 300 days, with a low OLR of 4.5 gVS/L/d and HRT 18 days (Figure 4A). Both fermenters generated 254 mL_STP_ CH_4_/gVS/L/d. Remarkably, at the low OLR of 4.5 g/L/d, the thermophilic fermenters F2 and F6 both showed higher methane yields than the mesophilic fermenter F1 and F3, with 224 and 227 mL_STP_ CH_4_/gVS, respectively (see the first column in Table 6 and Table 7). The highest methane yields of the mesophilic fermenter F1 and F3 were observed at a medium OLR of 7.5 gVS/L/d and after supplementation with nickel, then later with tungsten. F1 and F3 achieved a specific GPR of 230 and 242 mL_STP_ CH_4_/gVS, respectively. However, the yields of the thermophilic fermenters F2 and F6 continuously declined, both from 254 to 179 and 182 mL_STP_ CH_4_/gVS, respectively. This occurred in the subsequent fermentation period with an OLR 7.5 gVS/L/d (Table 6 and Table 7). It seemed that the hydrolysis of straw was complete at a low OLR, but this was more pronounced under thermophilic conditions. However, without the supplementation of nickel and tungsten, the specific GPR in the mesophilic, fermenter F4 decreased, finally, to 148 mL_STP_ CH_4_/gVS, and that of the thermophilic reference fermenter F5 decreased to 139 mL_STP_ CH_4_/gVS (Table 6 and Table 7). Additionally, the VFA production and resulting level of VFA were negatively affected by the absence of nickel and tungsten in the reference fermenters. They showed the highest VFA levels, with 5000–9000 (fermenter F4) and 3000–6000 (fermenter F5) at the end of the fermentation campaign after 640 days (Figure 4A, B, Table 6 and Table 7, column 4). The initial strategy was to induce trace elements requirement, by increasing the OLR from 7.5 (HRT of 10 days) to 9.0 gVS/L/d (HRT of 8 days). However, this led to indications of instability (Figure 4A, period 4 from 440–570 d), as the VFA increased from 300 to 4000 in the mesophilic fermenter F1 and from 2000 to 6000 mg/L in the thermophilic fermenter F2. The VFA concentration in the mesophilic reference fermenter F4, without supplementation, was between 5000–9000 mg/L, whereas the VFA concentration in the thermophilic reference fermenter F5 ranged between 3000–6000 mg/L after 640 d (Table 6 and Table 7).

However, the pH was stable between 7.3–7.6 and cell counts still increased between 440–570 days (Table 5, sampling time 423 and 521 d). It showed that the sensitive VFA level alone should not be indicator of process imbalance. The buffer capacity should also be considered. However, the buffer capacity is a very slow indicator [10]. Furthermore, after adding tungsten in the last fermentation period 5, the specific GPR increased in the mesophilic fermenter F1 from about 0.155 to 0.230 L/gVS, whereas it slightly increased in the thermophilic fermenter F2 from 0.146 up to 0.179 L/gVS (Figure 4A). Thereby, as expected, the VFA concentration decreased from 4000 to 1000 mg/l in the mesophilic fermenter F1 and decreased from 6000 to 2000 mg/L in the thermophilic fermenter F2 (Table 6 and Table 7, column 4).

However, successive addition of nickel alone during fermentation periods 1–4 in mesophilic fermenter F1 and thermophilic fermenter F2 (Figure 2) did not cause any increase in methane production, if compared with Figure 4A. This is somewhat in contrast to the slow increase of methanogenic cells of Figure 2 and Table 5. However, an obvious increase in specific GPR occurred after tungsten was added in period 5 (Figure 4A, Table 7). The GPR increase was directly related to a huge increase of the number of methanogenic cells by 200–300% (Figure 2 and Figure 3 and Table 5), but only to a low or moderate rise of total cell counts. It seems that the limited number of methanogenic cells in the range of 10^8^ mL^−1^ was a ‘bottleneck’ for the maximum possible methane production, as well as for VFA-consumption (mainly acetic acid, >80%).

Besides the methanogens, hydrolytic and syntrophic acetate oxidizing bacteria could be limiting in the anaerobic food chain [41,42]. The much lower increase of total bacterial cell counts (Table 4 and Table 5) could be an indication, that the presence of syntrophic bacteria was already adequate. This somewhat contradicts the old dogma of the rate-limiting step of polymeric carbohydrate degradation by hydrolytic bacteria (and not methanogens), as shown with cellulose and dissolved starch, glucose, and acetic acid [43]. Notably, in the case of straw, the cellulose biodegradation is much more recalcitrant for hydrolytic bacteria, as it is additionally complexed by lignin [44].

A somewhat different situation, with respect to VFA levels and methane production, was seen after fermentation, day 315, during the combined supplementation of nickel and tungsten in F3 (mesophilic) and F6 (thermophilic), with the constant OLR of 7.5 gVS/L/day after 315 d (Figure 3 and Figure 4B). There was a permanent increase in the volumetric biogas production (L/L fermenter volume FV) from F3 by 23% to about 3.7 L/L/d and 16% in F6 to about 2.8 L/L/d (Figure 4B, Table 6 and Table 7). As outlined under Figure 3, 0.05 mg W/L was first added in period 1 (370–405 day); after that, a combination of 0.05 mg/L [Ni + W] and EDTA was added to both F3 and F6 in period 2 (405–540 day). Then, finally, an increased concentration of 0.15 mg/L [Ni + W] + EDTA was added to both fermenters F3 and F6 in period 3 (540–640 day). As shown in Figure 3, the supplementation caused a sudden increase in the dissolved concentration of nickel and tungsten to reach 0.125 mg/L in fermentation period 3 (Figure 3). That was accompanied by an obvious increase in the specific GPR, as well by the number of methanogenic cells, which increased only in this period after tungsten was added. It seems that a concentration of dissolved tungsten (0.05 mg/L) was insufficient and, thus, indicated a threshold concentration of about 0.1 mg/L dissolved tungsten was necessary to reach the optimum for space-time methane yields and low VFA levels (Figure 4B). As already shown, the increase of the specific GPR/gVS was accompanied by a permanent simultaneous decline of the VFA-level in the mesophilic fermenter F3, even from 2000 mg/L to 200 mg/L. A similar VFA decrease was seen during successive supplementation of nickel and later tungsten in the mesophilic fermenter F1 (Figure 2 and Figure 4A, Table 6 and Table 7). The VFA level in the more sensitive thermophilic fermenter F2 and F6 declined only from 6000 or 4000 to ± 2000 mg/L (Table 7). The composition of VFA in all fermenters was similar and consisted mainly of acetic acid (>80%), propionic acid (<15%), and a lesser amount of butyric acid (2.5%). Table 6 and Table 7 show a summary of average values for maximum specific GPR/gVS, together with the average VFA levels of all six continuously stirred straw fermenters with buffered ‘synthetic manure’.

### 3.4. Comparison of the Methane Yields of Wheat Straw in this Work with Yields from Literature

A recent study on batch fermentations, supplemented with trace elements and ‘synthetic manure’ in our lab, showed a mean specific methane yield of 287.1 mL_STP_ CH_4_/gVS for the same charge of wheat straw used here, which corresponded to 98% of the theoretical methane yield [22]. This theoretical methane yield, based on the extended Buswell formula of straw (C_3.71_H_6.04_O_2.79_N_0.044_S_0.005_,) in Table 1, was calculated to be 293.4 mL_STP_ CH_4_/gVS. However, it is not possible to achieve 100% methane gas yields under practical conditions, because up to 8% of the carbon is assumed to be used in a BMP assay for production of new microbial biomass [45]. The maximum methane yield of 254 mL_STP_ CH_4_/gVS, found in this study with continuously stirred tank reactors (Table 6 and Table 7), in reality represents more than 88.5% of the maximum yield, based on the Buswell equation [23]. However, the methane yields of continuously stirred lab fermenters are always somewhat lower than the yields of BMP assays, because some of the substrate is lost through a narrow by-pass of the substrate inlet and fermenter outlet, which cannot be prevented in a small laboratory-scale set up. Such a bypass-loss is negligible in pilot or full-scale biogas plants. Therefore, a more than 90% straw conversion must be assumed here in the CSTR system. The high degradation rate, again, confirmed the suitability of ‘synthetic manure’ for biogas fermentations, as well as the concept of a buffered system with a stable pH between 7.3–7.6 and enriched with about 0.1 mg/L of the necessary trace elements in its dissolved, active form [22].

Currently, there are only few publications on the anaerobic degradation of wheat straw in continuously stirred fermenters, as performed here without any special thermal or chemical pre-treatment. For example, the authors in [46] investigated biogas production from wheat straw, by using 4-L laboratory CSTR fermenters filled with 3 litres of inoculum source. They monitored the system for a period of only 60 days and improved their methane production from wheat straw by recirculating some of the reactor effluent. It seems worth mentioning that the recirculation of 5–10% fermenter effluent through a by-pass system is already a practice in full-scale biogas plants (without liquid manure) in Germany, but generally to adjust the viscosity of the substrate to make it pumpable.

The same authors mentioned before [46] obtained an average methane yield of 240 mL_STP_ CH_4_/gVS at an OLR of 2 gVS/L, but with a long HRT of 40 days. However, the authors presumably did not standardize the gas volume at 0 °C and atmospheric pressure. This would result in an increase of the methane yield by about 10%. Other authors [47] studied the anaerobic degradation of wheat straw, but in 8-L laboratory scale continuous stirred fermenters containing 5 litres of an inoculum from a full-scale agricultural biogas plant. The fermenters were manually fed once daily for 6 days in a week. Unfortunately, the fermentation period was only 90 days, at 37 °C, with an OLR of 2.8 gVS/L/d and HRT of 26 days. The methane yield ranged between 130–210 mL _STP_ CH_4_/gVS. In addition, authors [48] worked with shredded and briquetted wheat straw in 15 L fermenters, filled with cow manure as inoculum and fermentation medium, for a period of 64 days at 50 °C. The fermenters were manually fed once daily for 5 days in a week, with an HRT of 25 days. They obtained methane yields of 214–217 mL _STP_ CH_4_/gVS [47].

Furthermore, authors [49] worked with a mesophilic 35 °C CSTR system, but fed in a batch mode using milled rice straw and pig manure as substrates. The authors simplified the conditions by using either the OLR 140 or 160 gTS/L over a period of only 50 and 60 days, which showed methane yields of 200–235 mL _STP_/gVS (presumably unstandardised gas volumes). Another novel reactor type was used by [50]. They worked with a solid up-flow-bed straw digester (39 L) at 60 °C, coupled with an anaerobic filter, with a volume of 30 L and recirculation of 1.15 L of effluent per hour. The study lasted for a period of 220 days, and they obtained increasing methane yields from 144 to 201 mL at simultaneously decreasing OLR, from 8 to 2.5 gVS/L/d. Apparently, their thermophilic system did not tolerate the same OLR of 7.5 gVS/L/d, similar to the results found here for the thermophilic fermenter F2 and F6, presented in Table 7. This highlights the new insight, that thermophilic fermentation of straw should not be performed above an OLR of 5 gVS/L/d. In summary, the findings in literature indicate that fermenters filled with large volumes of inoculum or of cow dung may not produce methane yields that match the yields obtained with ‘synthetic manure’, which was supplemented by nickel and tungsten, as presented here (Table 6 and Table 7). The results showed that, for a long-term fermentation with an automated, intermittently-fed CSTR system, the adapted microbiology worked quite optimally, without the need for any effluent recirculation or an extra thermal/chemical pre-treatment of wheat straw.

### 3.5. Considerations about the Right Trace Element Supplementation for Biogas Plants

As mentioned above, a threshold concentration of about 0.1 mg/L dissolved tungsten ions was necessary. Furthermore, the requirement of nickel and tungsten was not modulated by the OLR, as lignocellulosic wheat straw contains very low concentrations of them or below detection limit (Table 1). Instead of OLR, the HRT influenced the requirement of trace elements, after washout during continuous fermentation, as demonstrated in the disappearance of the nickel peak with an HRT of 18 days. The washout of nickel needed 290 fermentation days (140 days start-up phase + 150 fermentation days, Figure 2 and Figure 3, see Section 2.5). Therefore, the long-term fermentation exhibited that, with stationary batch tests, as sometimes claimed, the necessary trace element requirements of a substrate or a substrate-mixture cannot be determined.

The dependence of biogas production on nickel is well known [5,6,7], but has not yet been shown for straw as a mono-substrate. Hitherto, a distinct, sole tungsten dependence, has only been known from pure H_2_-CO_2_-utilising methanogenic cultures [19], but not yet from mixed biogas cultures, as outlined in the introduction. Astonishingly, the presented optimum concentrations for dissolved ions nickel and tungsten, of approximately 0.1 mg/L, lay in the same range of 1 µmole/L Co, Ni, and Mo, as reported for pure cultures of methanogens in aqueous media. As Co, Ni, and Mo have molecular weights of 59, 58.7, and 96, respectively, the commonly found optimum concentrations of 1 µM for trace elements [6] are equivalent to 0.06–0.1 mg/L, as found for the straw digestion with ‘synthetic manure’. Such data for pure cultures were summarized by [6] and included early data of methanogens, in addition to *Methanosarcina* with 1 µM or nearly 0.06 mg/L cobalt [51]. These concentrations were also found for bacterial pure cultures, see list for microbial culture media (www.dmsz.de, accessed on 15 July 2021). Notably, while studying a special *E. coli* strain, the authors [20] recently found that 1 µM tungsten in the growth medium, as applied here, can replace the bound molybdenum in a formate converting enzyme complex by tungsten, which shifted the catalytic bias of formate splitting to CO_2_ and H_2_, toward the reverse “hydrogenotrophic” reduction of CO_2_ with H_2_ to formate as the main fermentation product. Accordingly, there exists the hypothesis that tungstoenzymes are better tuned to reactions that proceed at more negative redox potentials, such as the carbon dioxide reduction with H_2_ at <−0.4 Volt, whereas the reverse formate oxidation is favoured by a more positive potential of molybdoenzymes between −0.2 and −0.4 V [52,53]. As presented already (see Section 3.2.2), the microbial numbers and 7.3% TS-content of the CSTR system and full-scale biogas plants are of the same order. It follows that the trace element requirement of any anaerobic digestion system could be more precisely and quickly evaluated, if the dissolved, active ions would be estimated. It would reduce the empirical, fastidious assessment process, based on process control parameters, such as monitoring volatile fatty acids in biogas plants for several months [12,13].

### 3.6. Microbial Community Composition

**Figure 5:**
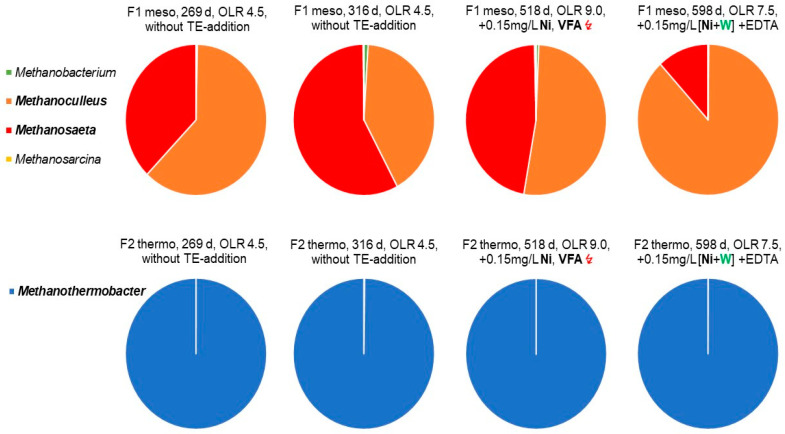
Taxonomic profiling of archaeal communities residing during long-term straw fermentation in continuously stirred laboratory fermenters, based on high-throughput 16S rRNA gene amplicon sequencing at different loading rates and successive nickel/tungsten supplementation. Relative abundances of the most abundant genera of archaeal subcommunities are shown. Complementary, graphical counterpart to Figure 2, with the number of methanogenic cells in fermenter F1 (mesophilic, 41 °C) and F2 (thermophilic, 58 °C).

The distinct effect of tungsten is known from studies with a pure culture of *Methanocorpusculum* grown mesophilic on H_2_-CO_2_ [19] and later from biochemical properties, as reviewed by [15,18]. *Methanocorpusculum* belongs to the order *Methanomicrobiales* like *Methanoculleus,* Figure 5. However, apparently, hydrogenotrophic species of the thermophilic genus *Methanobacterium* also require tungsten [17]. Furthermore, tungsten dependence of fermentative bacteria is also indicated, mainly by biochemical data [18,20].

Figure 5 shows the NGS-data for methanogens. *Methanothermobacter* was found to be exclusively dominant in the thermophilic fermenter F2 on day 269 d. The thermophilic genus *Methanothermobacter* belongs to the class of *Methanobacteria* and exhibited a relative abundance of 6–22%. However, the microscopic quantification (Table 4 and Table 5) showed that methanogens represented only 4–9% of the relative abundance. Such a divergence is not unusual, as the NGS-data are only qualitatively exact and manipulated by activities such as DNA extraction, choice of the right primer, PCR amplification, and, last but not least, the used software program of the database used [54]. A low proportion of methanogens, in the range of 5–10%, is also not unusual for complex substrates with a high percentage of hydrolytic bacteria. It reflects a high activity and functionality of the anaerobic community, as presented in other studies, too [31,32,55,56].

*Methanothermobacter* is a methanogen that uses only H_2_ and CO_2_. It is mostly found to be dominant in agricultural-based, thermophilic biogas plants [57,58]. This occurrence corresponds to the general assumption that a high substrate load and/or thermophilic conditions leads to an increase in partial hydrogen pressure and extends the energetically more favourable thermophilic H_2_-CO_2_ consumers [32,58]. The successive addition of nickel, after 340 d and tungsten on 570 d, did not change the sole dominance of *Methanothermobacter* (Figure 5), but it increased the cell number of methanogens tremendously (Figure 2, Table 5).

In contrast to thermophilic conditions, the NGS analysis of mesophilic archaea revealed a 3–7% abundance of the class *Methanomicrobia*, including the order *Methanomicrobiales*, with the genus *Methanoculleus*, furthermore, the order *Methanosarcinales*, with the genus *Methanosaeta*, as the most dominant representatives (>1%, pie charts of Figure 5). Currently, the genus of *Methanoculleus* comprises a group of at least 12 species, with a typical diameter of >2 µm and exclusively H_2_/CO_2_ consumers. In addition, some methanogens use formate as substrate [59], not found here. The genus *Methanosaeta* is a typical acetotrophic methanogen and metabolizes acetate as sole source of energy. It comprises of four species [59].

As shown in the top row of Figure 5, for the mesophilic fermenter F1, the relation of the genera *Methanosaeta* and *Methanoculleus* was with 55: 45% at the beginning of the fermentation campaign on day 269 d and changed slightly to 60: 40% on fermentation day 316 d. However, the mesophilic fermenter F1 was first supplemented with a small amount of nickel (0.05 mg/L) in fermentation period 3 (405–440 day), whereas the thermophilic fermenter F2 had started to supplement the same amount of nickel already in the previous fermentation periods 1 and 2 (340–405 day), Figure 2. In fermentation period 4 (440–570 day), the added amount of nickel was tripled, together with EDTA, to improve the availability of the bivalent nickel ions. At the end of the monitored fermentation campaign, that is after the successive addition of nickel in four periods (340–570 day) and final addition of tungsten in period 5 (after 570 day), the methanogenesis in the mesophilic fermenter F1 was clearly shifted in favour of the H_2_-CO_2_ consuming *Methanoculleus* with 85: 15% over *Methanosaeta*. However, after nickel supplementation, between 405 d and day 518 d (Figure 2), a slight dominance of *Methanoculleus* over *Methanosaeta* could be recognized, increasing from 45% to about 55% (Figure 5). This was accompanied by a slight increase of methanogens and total cell counts (Table 4 and Table 5). However, the sensitive VFA-level arose in this fermentation period 4, remarkably from 100–300 to 1000 to 4000 mg/L, as caused by the increased OLR of 9.0 gVS/L/d, with the reduced HRT of 8 days (fermenter F1, Table 7). The threefold VFA level might also result in a raise of the partial H_2_ pressure, in favour of hydrogenotrophic methanogens, such as *Methanoculleus*. However, the final decrease of VFA in period 5 down to 1000 mg/L occurred only after tungsten addition (Table 7). Additionally, after final tungsten supplementation, the number of methanogenic cells jumped abruptly by a factor of 330% (Figure 2 and Table 5). Coincidently, the proportion of *Methanoculleus* increased by tungsten from 55% to 90% on fermentation day 598 d (period 5), and at the same time, the portion of *Methanosaeta* decreased to about 10% (Figure 5). Therefore, it can be concluded that, during the sudden rise in the number of methanogenic cells, the hydrogenotrophic genus *Methanoculleus* replaced the acetotrophic genus *Methanosaeta*. Consequentially, the methanogenesis must have switched from acetotrophic (*Methanosaeta*) to hydrogenotrophic growth (*Methanoculleus*), Figure 5. It is noteworthy that only H_2_-CO_2_ consuming methanogens need the CO_2_ activating formylmethanofuran dehydrogenase, which could be a constitutive tungsten enzyme [15,18]. However, apparently, a threshold of about 0.1 mg/L dissolved tungsten was required, as revealed independently by the two different OLR regimes with successive (Figure 2) and combined supplementation (Figure 3). Parallel, the specific GPR in the fermenter F1, F2, F3, and F6 increased after tungsten supplementation and induced a simultaneous reduction of the VFA level (Table 6 and Table 7). Meanwhile, the specific GPR of the un-supplemented reference fermenter F4 and F5 continuously dropped down and remained at a modest level, but with a high VFA level of 6000–9000 mg/L (Table 6 and Table 7).

Overall, it seems worthwhile to emphasize that the study demonstrated trace element dependency, only after a very long time of fermentation. The monitoring of dissolved trace elements during the fermentation campaign revealed that 290 days are needed for complete washout of the nickel-peak of inoculum (Figure 2 and Figure 3, Table 3; 140 d pre-fermentation period and 150 d fermentation with an HRT of 18 d, see Section 2.5). Therefore, contrary results with no increase in the number of methanogens by trace element addition during fermentation of grass silage for only 70 days could be due to insufficient washout of trace elements [60,61]. In their study [60,61], the supplemented trace elements (Co, Fe, and Ni) were added in concentrations of around 10 mg/L, being at least 100 times higher than the optimum concentrations found in this study with wheat straw and ‘synthetic manure’.

Similar experiments, studying the contribution of methanogens during biogas production in laboratory fermenters, were also conducted by [62]. The authors investigated the varying relationship between *Methanosarcina* and *Methanoculleus* during methanogenic fermentation of the protein-rich leftover of an alcoholic yeast fermentation, referred to as ‘distillers grain’ [62]. They monitored the process for a period of 532 days (76 weeks), in order to investigate the influence of a suggested cocktail of trace elements (a mix of Co, Mo, Mn, and W). The initial deprivation of the system of trace elements resulted in a decline of Co and Mo, from 2.9 and 0.8 mg/L to 0.9 and 0.2 mg/L, respectively. Mn decreased from 38 to 1.8 mg/L and W decreased from 1.4 to 0.2 mg/L [61]. This was a high washout, but presumably not enough to provoke a deficiency, if compared with the optimum concentrations found in the present work and for pure cultures of methanogens, in the range of 0.1 mg/L, as summarized by [6]. Accordingly, after their washout period, the authors of [60] measured only a slight drop down of 5% by *Methanosarcina*, from 72% to 67% and a concomitant increase of *Methanoculleus*. Therefore, the results in [61], with a much higher concentrated mix of at least 4 elements (including tungsten), cannot be compared with the results of this study.

**Figure 6:**
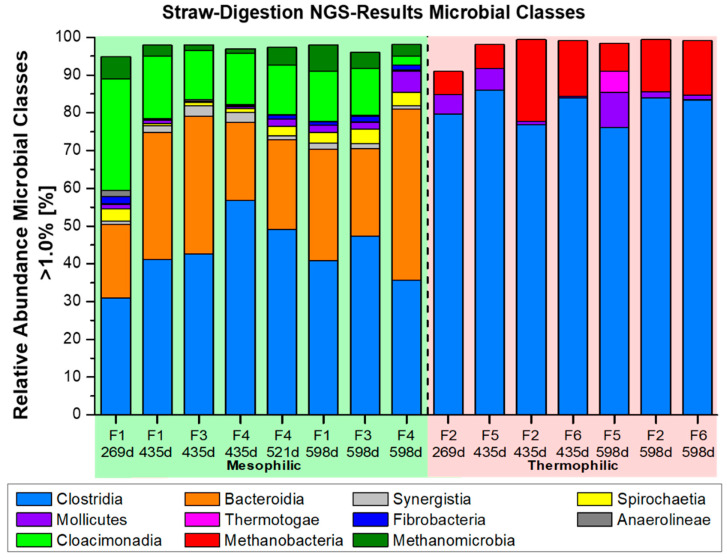
Relative abundance of the most important microbial classes, with an abundance of >1.0% (major players), which were recorded by NGS analysis in the different mesophilic and thermophilic wheat straw fermenters F1–F6 (Figure 1B) with ‘synthetic manure’ as defined medium.

**Figure 7:**
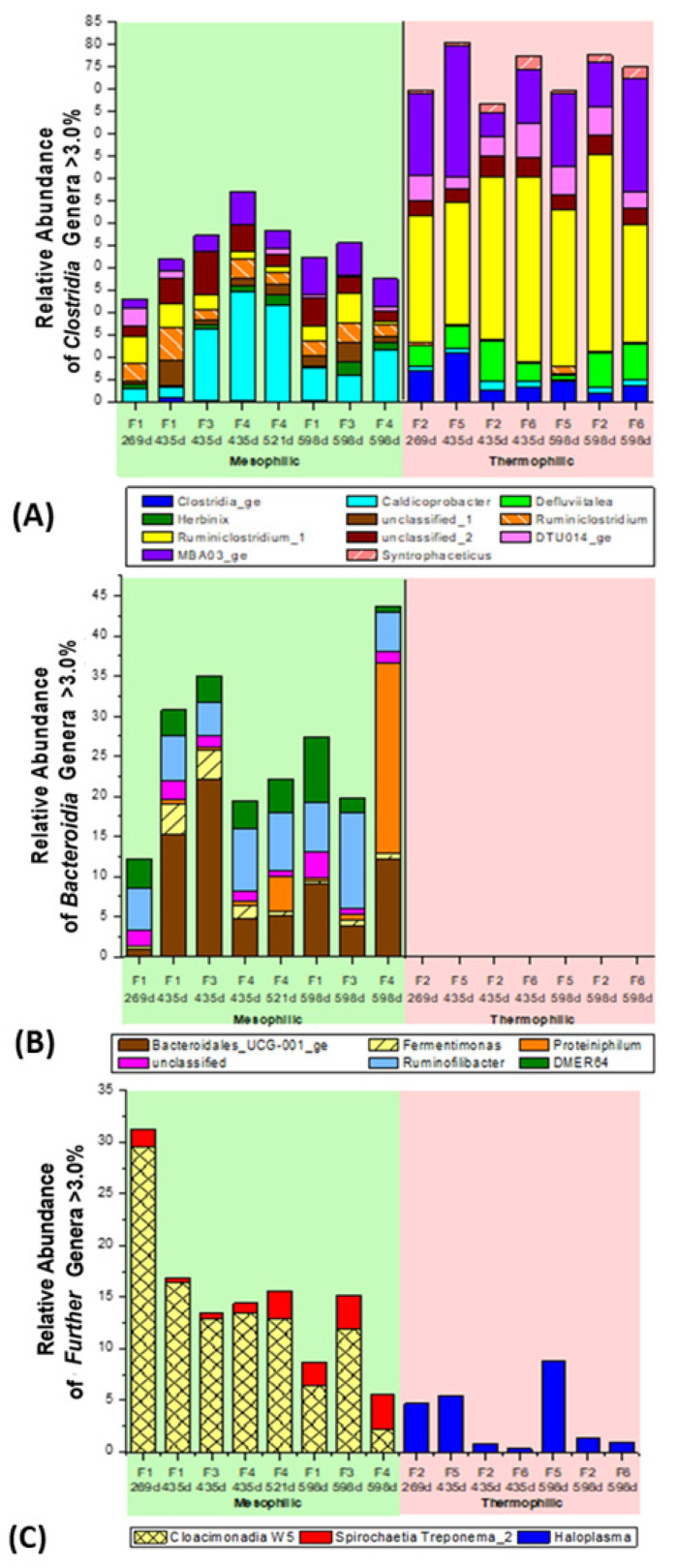
Relative abundance of the dominant microbial genera, with an abundance of >1.0% or 3% (major players), which were recorded by NGS analysis in the different mesophilic and thermophilic wheat straw fermenters F1–F6 (Figure 1B), with ‘synthetic manure’ as defined medium. (**A**) *Clostridia* Genera > 3%, (**B**) *Bacteroidia* Genera > 3%, (**C**) *Further* Genera > 3%.

Additional NGS-microbiome data, with respect to the involved major players of bacterial classes and genera, have been presented in Figure 6 and Figure 7A–C. Their occurrence will be compared with the related literature data regarding the anaerobic digestion of lignocellulosic substrates. The percentage of classes with a relative occurrence of less than 1% is extremely low, both in the mesophilic microbial communities and, especially, in the thermophilic composition. Thus, the sum of the classes that occur more than 1% is over 90%. The mesophilic fermenters F1, F3, and F4 exhibited a spectrum of 7 relevant bacterial classes with >1% abundance, containing 17 major bacterial genera in the genus list >3% abundance. The thermophilic fermenters F2, F5, and F6 were mainly restricted to only two dominant classes >1%, but with 11 major bacterial genera in the genus list >3% abundance. Such a dominance of only two bacterial, thermophilic classes (*Clostridia* and *Mollicutes*) in the thermophilic fermenter F2, F5, and F6 is quite unique and not previously found in literature related to methanogenic fermentation of biomass. The genera *Syntrophaceticus*, *Defluviitalea*, *Ruminiclostridium*, and *Haloplasma* were observed entirely in thermophilic fermenter F2, F5, and F6, whereas the bacterial genus *Herbinix* and uncultured genus ‘UCG_001_ge’ appeared only in the mesophilic fermenter F1, F3, and F4. It is noteworthy that a total of 285 mesophilic and 167 thermophilic genera <1% were identified through NGS-analysis. Thus, a high bacterial diversity still existed as a gene reservoir in the background, but a strong selection must have occurred in this long-term, CSTR fermentation with straw under defined fermenter conditions and ‘synthetic manure’ (Table 2).

The bacterial class *Clostridia* of the phylum *Firmicutes* (bright blue) was dominant in the mesophilic, as well as in the thermophilic fermenters. *Clostridia* represented a contingent of 30–55% of the >1% abundant classes at the beginning of the supplementation period (45 d) and continued over the whole fermentation period, until the last sample of fermentation day 598 d (Figure 6). However, this outstanding dominant contribution by the clostridial *Firmicutes* during mesophilic straw fermentation was even surpassed in the thermophilic fermenters, with about 75–85% abundance. *Firmicutes* also dominated in other thermophilic biogas fermenter system with wheat straw as the sole substrate, as investigated by [63,64]. In contrast, the class *Bacteroidia* (phylum Bacteroidetes, orange) occurred only under mesophilic conditions in fermenter F1, F3, and F4. They gained importance and reached up to 45% abundance in the classes with >1% abundance. *Firmicutes* and *Bacteroidetes* are known to be involved in the hydrolytic degradation of cellulosic compounds. Hence, they were found to be dominant in agricultural-based biogas plants or biogas fermenters fed with cellulosic compounds [55,56,58,65,66,67,68,69]. Both phyla, *Firmicutes* and *Bacteroidetes*, can be exchanged with each other, as shown by [57], in a long-term fermentation period of 1750 days, with fodder beet silage as a mono-substrate. Interestingly, in this work they were not major players right from the beginning, as they first appeared in the sample on fermentation day 269 d (Figure 6). Notably, also the dominant occurrence of the class *Cloacimonadia* (bright green) was restricted to the mesophilic fermenters, just as the *Bacteroidia* (Figure 6). They presented major players of straw digestion, with up to 30%, as shown in Figure 6. The outstanding genus candidatus ‘W5 group of *Cloacimonas*’ in the mesophilic fermenters will be discussed later, with respect to further relevant genera. The class *Synergistia* (grey) decreased during the entire fermentation period, from nearly 20% to 2–3%. *Synergistia* are sugar fermenting bacteria and consist largely of different *Anaerobaculum* species, synonymous with *Acetomicrobium* [70]. In a recent paper of a combined enzymatic and anaerobic digestion process, with the lignocellulosic substrate “cedarwood”, the *Synergistia* belonged to the four most relevant classes, making up 6–44% relative frequency, besides *Methanobacteria*, *Clostridia*, and *Thermotogae*.

The bacterial phyla of the thermophilic fermenter F2, F6, and reference F5 seemed to be more restricted or specialized than the phyla of the mesophilic fermenter F1, F3, and F4. Only two dominant bacterial groups, the class *Clostridia* (bright blue, phylum *Firmicutes*) and osmotolerant *Mollicutes* (violet, phylum *Tenericutes*) were found, under thermophilic conditions, to account for 1–9% of the classes, with an abundance > 1%, besides the archaeal methanogens. However, *Mollicutes* were also recognized in the mesophilic fermenter F1, F3, and F4 (Figure 6). Furthermore, *Mollicutes* have been observed in some agricultural-based biogas plants [58] and a thermophilic biowaste digester [56]. The clostridial *Defluviitalea* belongs to the *Mollicutes* and was found to be present here, with 4–14% as the sole thermophilic genus of this group with an abundance >3% (bright green, Figure 7A). This fermentative genus, with two different species, is able to grow on carbohydrates and was already observed in a rice straw fermenter [71].

As already mentioned, no major role of the class *Bacteroidia* >1% was noticed under the thermophilic conditions. On the other side, *Thermotogae* (>1% abundance) are well known from thermophilic biogas plants [31,56]. However, here, they were found to occur only in the thermophilic, unsupplemented reference fermenter F5 on fermentation day 598 d (Figure 6). The classes *Clostridia*, *Mollicutes*, and *Bacteroidia* played a relevant role (>1% abundance) for both mesophilic and thermophilic straw fermentation. The Mollicutes accommodate the order *Haloplasmatales*, with *Haloplasma* as the sole genus. The exclusive appearance of the genus *Haloplasma* was seen in the list of relevant players >3%, with an abundance up to 9% (Figure 7C). However, this genus was only related to the thermophilic fermenter F2, F5, and F6 (Figure 7C). However, the occurrence of *Haloplasmatales* in anaerobic biomass digesters has been rarely observed. The authors [72] found them in 1.5-L laboratory biogas fermenters (55 °C, 200 days), using lignocellulosic switch grass as the mono-substrate. The genus *Haloplasma* has no cell wall and was also seen as a major player in thermophilic 100 mL biogas batch assays at 55 °C, but with different kinds of paper-cellulose as the substrate [73]. Interestingly, the clostridial *Halocella* is an osmotolerant bacterium similar to *Haloplasma*, too, but with a cell wall. *Halocella* was observed to be a major player in full-scale, thermophilic biogas plants, as well [31,56,65,72]. Seemingly, *Haloplasma* could replace *Halocella* in thermophilic anaerobic digesters with cellulosic material. This finding is quite unique and not previously found in literature. It may be an indication that the ‘synthetic manure’, with a similar osmolarity to natural cow manure (Table 2), forced osmotolerant genera in the same manner.

Another outstanding detection of a representative of the *Cloacimonadia* was the W5 group of *Cloacimonas*, with up to 29% occurrence in the list with >3% abundance of mesophilic genera (Figure 7C). The order *Cloacimonadales* was just recently found as a major player in different mesophilic agricultural-based biogas plants, by [69]. However, another genus of this group was found to be relevant in a thermophilic agricultural-based biogas plant [55]. The authors [55] designated it as genus ‘*Cloacimonetes* WWE1’. Possibly, *Cloacimonadia* plays an important role as propionate oxidizers. Some members of Cloacimonetes are known to be propionate oxidizers [73], which live in obligate syntrophy with hydrogenotrophic methanogens [41]. Their presence could explain that the increase of VFA during the slight imbalanced states of the fermentation campaign was restricted, mainly due to acetic acid. Only a minor part <15% propionic acid contributed to the VFA level (Table 6 and Table 7).

Another remarkable finding was the discovery of the mesophilic, cellulolytic genus *Ruminiclostridium*_1, with 28–44% occurrence (yellow) in the thermophilic fermenters. However, this genus occurred to a smaller extent (5%), as well as in all mesophilic fermenters (Figure 7A). The authors [26], with their 100 mL batch straw assays, as well as the authors [69], with their study of 20 full-scale agricultural biogas plants, found this genus as a major player, too.

The high frequency of the clostridial cluster genus MBA03_ge, with 6–29% abundance in the list of the major genera with >3% abundance, was also outstanding. MBA03_ge was recently reported to play a dominant role, with up to 21% abundance in the anaerobic degradation of lignocellulosic grass [61] and during anaerobic degradation of a typical municipal thermophilic biowaste, including assorted lignocellulosic garden and vegetarian kitchen residues [56]. Our discovery of this genus was more a coincidence. First, this novel genus was not detected in all samples, as the Silva database nr119 (from 2017) was applied (see Section 2.4). However, the re-assessed informatics evaluation of the same molecular sample, with the more updated Silva database nr132 (version from 2019), exhibited the unexpectedly high abundance of MBA03_ge, as seen in Figure 7A.

Furthermore, the list of major players (>3%) for straw digestion could be continued, with the abundance of 3–25% by the mesophilic *Caldicoprobacter* and up to 7% by the unclassified Clostridium genus DTU014_ge. Additionally, the syntrophic thermophilic *Syntrophaceticus* played, with 3–9% contribution of the abundance list >3%, a major role (Figure 7A). Interestingly, these three genera, including MBA03_ge, were observed in a thermophilic municipal biowaste digester study, too [56]. According to the authors [56], the probable reason for the abundance of the three genera, during the long-term fermentation of biowaste, was the replacement of acetoclastic methanogenesis by syntrophic acetate oxidation, combined with hydrogenotrophic methanogenesis. This view coincides quite well with the parallel, predominant occurrence of the hydrogenotrophic genus *Methanoculleus* and *Methanothermobacter*, under both mesophilic and thermophilic conditions of straw digestion (Figure 5).

Another major player, observed under both fermentation temperatures, was the prementioned hydrolytic genus *Ruminiclostridium* _1 (yellow), as well as the related ‘R. spec’ (Figure 7A, B, orange), as also found by [26,73]. Similar to *Ruminiclostridium*, the hydrolytic *Ruminofilibacter* belongs to the hydrolytic class *Bacteroidia*/family *Rikenellaceae* [65] and made up to 12% of the major players here (Figure 7B). It also seems worthy to note that the dominant occurrence of the novel uncultured genus “UCG-001_ge”, which belongs to the order *Bacteroidales*, made up almost 25% of the genera with >3% abundance, but only in the mesophilic straw fermenters (Figure 7B).

As already mentioned, the hemicellulolytic *Herbinix* was found with high abundance in the mesophilic fermenter F1, F3, and F4 (Figure 7A, green). Its presence has also been reported in full-scale biogas plants, by [26,31,67] and in this work (Figure 7A–C).

Finally, saccharolytic, fermentative genera played a relevant role during the investigated long-term straw fermentation under CSTR conditions. Besides the unclassified genus of *Bacteroidia* UCG-001_ge, the saccharolytic genus *Fermentimonas* [31,49], fermentative genus *Proteiniphilum* [26,32,49,56,67,74], and novel genus ‘DMER64′ played a major role. The authors of [49] worked with a mesophilic (35 °C) batch system, using milled rice straw and pig manure as substrates. They found over 60% abundance of *Bacteroidetes* (phylum level) and about 40–55% abundance of this novel genus ‘DMER64′ [49]. In fact, ‘DMER64′ seems to be an excellent degrader of lignocellulosic biomass. Thus, a future microbiological characterisation study could validate the efficiency of this genus or its species.

Worth mentioning is the relevant occurrence of *Treponema*_2, belonging to the class *Spirochaetia* of the phylum *Spirochaetes,* because it fits in the category of a highly selected straw degrading population (Figure 7C). This genus was noted to degrade cellulosic biomass in full-scale anaerobic digesters [75] and the rumen of a red deer [76]. Additionally, some relevant genera failed to be assigned to known genera. For example, unclassified clostridial genera, designated as 1 (only mesophilic) and 2 (both meso- and thermophilic), were found to be major players, too (Figure 7A).

## 4. Conclusions

In both experimental scenarios of anaerobic straw digestion with different OLR strategies, either the successive addition of nickel and then, finally, adding tungsten or combined supplementation with increasing concentrations of Ni and W, led to similar results in the subsequent, final fermentation periods. A threshold concentration of approximately 0.1 mg/L tungsten ions seemed to be necessary. Tungsten caused a tremendous increase of microscopically counted methanogens, by NGS-data specifically related to hydrogenotrophic methanogens, such as *Methanoculleus* or *Methanothermobacter*. Tungsten also caused an increase in the volumetric and specific methane production GPR (mL_CH4/_g_VS_) and simultaneously provoked a decrease of VFA concentration. The specific GPR of the un-supplemented reference fermenter F4 and F5 dropped down continuously and remained on a low GPR and high VFA level. Continuous TXRF analysis of the dissolved, active trace element ions revealed that 290 days were required to completely washout trace elements from the inoculum, as shown in the timeline of the nickel peak. The nickel ion peak disappeared below detection limit, which was seen nearly exactly in all 4 supplemented fermenters after 290 d, mesophilic or thermophilic, as shown in Figure 2 and Figure 3. It is significant to note that the mesophilic fermenter F1, F3, and F4, with a low OLR of 4.5 gVS/L/day and HRT of 18 days, showed lower specific methane yields and, thus, lower degradation rates than the thermophilic fermenter F2, F5, and F6 at the same OLR and HRT. However, the performance of mesophilic fermentation was found to be superior at higher loading rates of 7.5 and 9.0 gVS/L/day, as validated by reduced VFA formation. Surprisingly, at high VFA levels (4000–6000 mg/L), cell numbers still increased. Perhaps this was a consequence of the stable pH, as a result of the buffered ‘synthetic manure’. The NGS-microbiome data gave no explicit answer to this phenomenon, because both microbial populations of the long-term straw digestion, mesophilic, as well as thermophilic, showed a very restricted and highly specialized microflora. Especially the thermophilic fermenters F2, F5, and F6 exhibited a restricted spectrum of only two relevant bacterial classes with >1% abundance, whereas the mesophilic fermenter F1, F3, and F4 contained seven dominant bacterial classes >1%. However, below 1% abundance, in a total of 285 mesophilic and 167 thermophilic genera were in the background. Several of the major bacterial players were attributed to be syntrophic or osmotolerant. Perhaps as a general principle, the determined optimum concentrations of dissolved ions nickel and tungsten lay in the same range of 1 µmole/L for Co, Ni, and Mo as that found with all pure cultures of methanogens on H_2_-CO_2_, acetate, or methanol (summarized by [6]). Such an optimum concentration is also used empirically for most of all cultured bacteria (see list for culture media www.dmsz.de, accessed on 15 July 2021). As outlined, the cell counts of pure bacterial cultures lie in the same range of 10^10^–10^11^ cells/mL, such as in agricultural biogas plants. Therefore, it is a challenging outlook that, with respect to trace element requirements, the biogas production behaves in the same manner as an aqueous fermentation. However, to estimate the trace element requirement, the analysis of dissolved, bioavailable ions should be introduced as a necessary prerequisite.

## Figures and Tables

**Table 1:** Composition of wheat straw, determined by total solids = dry weight (TS), volatile solids = organic dry weight (VS), wood sugar, and elemental analyses, as well as TXRF methodology (transmission X-ray fluorescence spectrometry).

Parameter	Wheat Straw	
Particle size [mm]	0.13	
Dry matter, TS ^1^ [%]	96.90	
Water Content [%]	3.10	
Organic dry matter, VS ^1^ [%]	90.10	
Ash content on ignition [%]	6.80	
Total carbon [%]	44.51	
Total nitrogen [%]	0.62	
C/N Ratio	84.30	
Total Hydrogen [% VS]	6.02	
Total Oxygen [% VS]	44.80	
Total Sulfur [% VS]	0.16	
Total Phosphorus [% VS]	0.09	
Cellulose ^2^ [%]	36.73	
Hemicellulose ^3^ [%]	25.26	
Lignin [%]	23.05	
Protein ^4^ [%]	3.40	
Fat ^5^ [%]	1.67	
Iron [mg/kg]	562 ± 20	RSD 3.62
Manganese [mg/kg]	35.01 ± 0.76	RSD 2.17
Zinc [mg/kg]	19.14 ± 0.63	RSD 3.29
Copper [mg/kg]	2.76 ± 0.11	RSD 3.99
Cobalt (Co) [mg/kg]	1.18 ± 0.03	RSD 2.54
Nickel (Ni) [mg/kg]	0.58 ± 0.04	RSD 6.89
Molybdenum [mg/kg (ICP-OES)	1.4	
Selenium (Se) [mg/kg]	BDL	
Tungsten (W) [mg/kg]	BDL	

^1^ See under process control parameters. ^2^ Cellulose a was calculated after high pressure liquid chromatography (HPLC) analysis of the glucose content [24]. ^3^ Hemicellulose was determined by the sum of xylose, arabinose, galactose, mannose, and rhamnose (HPLC analysis). ^4^ Protein = total Nitrogen × 5.46 (N estimated by elementary analysis). ^5^ Fat was regarded as the remaining part of VS, after all other biochemical constituents were estimated. Percentage elements C, H, O, N, S, P for the Buswell straw formula were with respect to VS. Trace element content with standard deviation RSD. BDL = below detection limit (<0.002 mg/L).

**Table 2:** Chemical composition of the mineral solution used as manure substitute and designated as ‘synthetic manure’ [22].

Chemical Salt Solution ‘Synthetic Manure’				Natural Cattle Manure ^1^
Composition		Main Ion	Weighted Salt	[mmol L^−1^]
(Salt, Gram-Molecular Weight)		[mmol L^−1^]	[g L^−1^]	
KHCO_3_	−100.115	50.1 K^+^	5.52	28.1–94.6 K^+^
K_2_CO_3_	−138.205	10.2 K^+^	0.705	
NH_4_HCO_3_	−79.055	138.6 NH_4_^+^	10.96	83.2–166.3 NH_4_^+^
CaCl_2_·6H_2_O	−219.076	6.2 Ca^2+^	1.36	3.0–11.2 Ca^2+^
MgCl_2_·6 H_2_O	−203.303	3.7 Mg^2+^	0.752	1.2–6.2 Mg^2+^
Fe_2_(SO_4_)_3_	−399.878	0.116 Fe^3+^	0.023	0.02–0.11 Fe^3+^
		0.174 SO_4_^2−^		2.6–3.7 SO_4_^2−^
Na_2_SO_4_	−142.042	0.20 Na^+^		
		0.10 SO_4_^2−^	0.142	
				17.4–52.2 Na^+^
NaHCO_3_	−84.007	30.9 Na^+^	2.596	

^1^ Chemical composition of cattle manure analysed over several years. The cattle manure was obtained from the biogas plant of a dairy farm in D-23845 Seth, Germany.

**Table 3:** Overview of relevant dissolved trace elements ions (Mn, Fe, Co, Cu, Zn, Ni, and W) during the monitored straw fermentation period of 640 days, as seen in Figure 1, Figure 2 and Figure 3. TXRF-analyses were performed every two weeks (in some periods weekly). Potassium and calcium ions (K, Ca) were chosen as a benchmark. BDL = below detection limit.

Element	Observation	Mesophilic Fermenter (41 °C)			Thermophilic Fermenter (58 °C)		
		F1	F3	F4 (Reference)	F2	F6	F5 (Reference)
	Concentration (mg/L)						
K	Min.	2247	2034	2090	2070	2202	2114
	Max.	2857	2791	3043	2850	2824	2807
	Average	2596	2461	2581	2499	2521	2460
Ca	Min.	85.7	91.8	87.9	33.4	31.9	33.1
	Max.	127.5	125.9	142.6	58.3	53.0	54.9
	Average	104.4	103.0	115.2	44.4	42.3	43.1
Mn	Min.	0.357	0.412	0.492	0.089	0.088	0.097
	Max.	0.779	0.680	0.699	0.099	0.116	0.126
	Average	0.594	0.571	0.603	0.095	0.098	0.114
Fe	Min.	7.70	6.13	8.06	6.39	6.07	6.15
	Max.	11.72	10.84	11.99	9.36	9.33	9.84
	Average	9.45	8.44	9.45	7.93	7.64	7.87
Co	Min.	0.071	0.106	0.140	0.122	0.128	0.108
	Max.	0.216	0.178	0.196	0.183	0.160	0.160
	Average	0.152	0.149	0.171	0.163	0.142	0.131
Cu	Min.	0.022	0.023	0.022	0.033	0.050	0.042
	Max.	0.065	0.076	0.063	0.086	0.092	0.098
	Average	0.038	0.049	0.037	0.052	0.068	0.071
Zn	Min.	0.021	0.059	0.029	0.043	0.043	0.039
	Max.	0.070	0.095	0.082	0.114	0.085	0.084
	Average	0.049	0.076	0.051	0.078	0.070	0.061
Ni		BDL	BDL	BDL	BDL	BDL	BDL
W		BDL	BDL	BDL	BDL	BDL	BDL

**Table 4:** Semi-automated cell counts by quantitative microscopy, during long-term anaerobic digestion of wheat straw, with ‘synthetic manure’ in the six fully automated and continuously stirred fermenters F1–F6. Data for only auto-fluorescent methanogenic cells.

Fermentation Day	Methanogenic Cell Counts					
	Mesophilic [Cells/mL]			Thermophilic [Cells/mL]		
	F4 (Reference)	F1	F3	F5 (Reference)	F2	F6
37 day	4.11 × 10^8^	3.46 × 10^8^	4.91 × 10^8^	4.03 × 10^8^	5.66 × 10^8^	4.89 × 10^8^
311 day	3.57 × 10^8^	2.48 × 10^8^	4.49 × 10^8^	3.61 × 10^8^	5.80 × 10^8^	5.15 × 10^8^
402 day	5.23 × 10^8^	3.46 × 10^8^	4.63 × 10^8^	4.33 × 10^8^	5.66 × 10^8^	4.75 × 10^8^
423 day		4.94 × 10^8^			6.52 × 10^8^	
521 day	5.21 × 10^8^	4.73 × 10^8^	5.34 × 10^8^	4.96 × 10^8^	7.21 × 10^8^	5.63 × 10^8^
570 day		5.14 × 10^8^			7.86 × 10^8^	
605 day		9.34 × 10^8^			9.09 × 10^8^	
622 day	6.54 × 10^8^	1.68 × 10^9^	1.28 × 10^9^	4.03 × 10^8^	1.58 × 10^9^	1.23 × 10^9^
640 day	5.32 × 10^8^	1.70 × 10^9^	1.76 × 10^9^	4.15 × 10^8^	1.53 × 10^9^	1.75 × 10^9^

**Table 5:** Semi-automated total cell counts, with a quantitative microscopy, during long-term anaerobic digestion of wheat straw, with ‘synthetic manure’ in the six fully automated and continuously stirred fermenters F1–F6. Total cell counts were obtained by staining with CyberGreen and included the methanogens.

Fermentation Day	Total Cell Counts					
	Mesophilic [Cells/mL]			Thermophilic [Cells/mL]		
	F4 (Reference)	F1	F3	F5 (Reference)	F2	F6
37 day	1.78 × 10^10^	1.38 × 10^10^	2.13 × 10^10^	1.75 × 10^10^	2.89 × 10^10^	2.12 × 10^10^
311 day	1.55 × 10^10^	1.08 × 10^10^	1.95 × 10^10^	1.56 × 10^10^	2.23 × 10^10^	1.83 × 10^10^
402 day	2.27 × 10^10^	1.38 × 10^10^	2.01 × 10^10^	1.88 × 10^10^	2.86 × 10^10^	2.07 × 10^10^
423 day		1.91 × 10^10^			2.42 × 10^10^	
521 day	1.86 × 10^10^	1.69 × 10^10^	2.32 × 10^10^	2.16 × 10^10^	2.58 × 10^10^	2.45 × 10^10^
570 day		1.82 × 10^10^			2.26 × 10^10^	
605 day		2.09 × 10^10^			3.29 × 10^10^	
622 day	2.28 × 10^10^	2.75 × 10^10^	2.20 × 10^10^	2.37 × 10^10^	2.69 × 10^10^	3.02 × 10^10^
640 day	2.10 × 10^10^	2.96 × 10^10^	1.86 × 10^10^	2.71 × 10^10^	2.72 × 10^10^	2.45 × 10^10^

**Table 6:** Specific methane production (spec GPR) of three fully automatic, continuously stirred mesophilic (41 °C) fermenters. They were fed with wheat straw, as the sole substrate, at different loading rates in ‘synthetic manure’ and supplemented with trace elements, according to Figure 2 and Figure 3. Reference fermenter F4 was not supplemented. All values were converted into mL under standard conditions of temperature and pressure and related to VS (thereby, approx. 10% reduced, with respect to raw gas measurement). The OLR/HRT-values of fermenter F3 were according to Figure 3. Therefore, column 4 refers to fermentation period 3 for F3 and not to period 5, as for F1.

Specific Methane Production from Wheat Straw in Mesophilic Fermenters F1, F3 and F4								
Fermenter	OLR 4.5, HRT 18 d		OLR 7.5, HRT 10 d		OLR 9.0, HRT 8 d		OLR 7.5, HRT 10 d	
	Spec. GPR (mL_CH4/_gVS)	VFA	Spec. GPR (mL_CH4/_gVS)	VFA	Spec. GPR (mL_CH4/_gVS)	VFA	Spec. GPR (mL_CH4/_gVS)	VFA
		(mg/L_FV_)		(mg/L_FV_)		mg/L_FV_		mg/L_FV_
F4 (−TE) ^1^	216 ^1^	<100	182 ^1^	100–1000	-	-	148 ^1^	5000–9000
		0–315 day		315–390 day				
								410–640 day
F1 (+TE)	224 ^1^	<100	205 ^2^	100–300	155 ^4^	1000–4000	230 ^5^	4000 decreased to 1000
			
		0–315 day		340–405 day		440–570 day		570–640 day
F3 (+TE)	227 ^1^	100–1000	206 ^3^	±1000		1000–2000	242 ^5^	2000 decreased to 200
								540–640 day
		0–315 day		380–410 day		410–540 day		

^1^ Without trace element addition TE; ^2^ +0.15 mg Ni L-1; ^3^ +0.05 mg/L [Ni + W]; ^4^ +0.15 mg/L Ni + EDTA; ^5^ +0.15 mg/L [Ni + W] + EDTA. OLR = gVS/L_FV_/d. FV = litre fermenter volume. Fermenter F1, F2; also see Figure 4. VFA: >80% acetic, <15% propionic and about 2.5% butyric acid.

**Table 7:** Specific methane production (spec GPR) of three fully automatic, continuously stirred thermophilic (41 °C) fermenters. They were fed exclusively with wheat straw at different loading rates in ‘synthetic manure’ and supplemented with trace elements. Reference fermenter F5 was not supplemented. All values are converted into mL under standard conditions of temperature and pressure and were related to VS (thereby, approx. 10% reduced, with respect to raw gas measurement). The OLR/HRT-values of fermenter F6 were according to Figure 3. Therefore, column 4 refers to fermentation period 3 for F6 and not to period 5, as for F2.

Specific Methane Production from Wheat Straw in Thermophilic Fermenters F2, F5 and F6								
Fermenter	OLR 4.5, HRT 18 d		OLR 7.5, HRT 10 d		OLR 9.0, HRT 8 d		OLR 7.5, HRT 10 d	
	Spec. GPR (mL_CH4/_gVS)	VFA (mg/L_FV_)	Spec. GPR (mL_CH4/_gVS)	VFA (mg/L_FV_)	Spec. GPR (mL_CH4/_gVS)	VFA mg/L_FV_	Spec. GPR (mL_CH4/_gVS)	VFA
								mg/L_FV_
F5 (−TE) ^1^	2481	<2000	158 ^1^	2000–4000	-	-	139 ^1^	3000–6000
		0–315 day		315–390 day				410–640 day
F2 (+TE)	254 ^1^	2000–2500	182 ^2^	2000–4000	146 ^4^	2000–6000	179 ^5^	6000 decreased to 2000
		0–315 day		340–405 day		440–570 day		570–640 day
F6 (+TE)	2541	1600	174 ^3^	4000 decreased to 1000		1000–2000	182 ^5^	±2000
		0–315 day		380–410 day		410–540 day		540–640 day

^1^ Without trace element addition TE; ^2^ +0.15 mg Ni L-1; ^3^ +0.05 mg/L [Ni + W]; ^4^ +0.15 mg Ni L^−1^ + EDTA; ^5^ +0.15 mg/L [Ni + W] + EDTA. OLR = gVS/L_FV_/d. FV = litre fermenter volume. Fermenter F1, F2; also see Figure 4. VFA: >80% acetic, <15% propionic, and about 2.5% butyric acid.

## Data Availability

The data presented in this study are available on request from the corresponding author.
